# Interventional radiological therapies in colorectal hepatic metastases

**DOI:** 10.3389/fonc.2023.963966

**Published:** 2023-05-30

**Authors:** Sai Swarupa R. Vulasala, Patrick D. Sutphin, Samira Kethu, Nirmal K. Onteddu, Sanjeeva P. Kalva

**Affiliations:** ^1^ Department of Radiology, University of Florida College of Medicine, Jacksonville, FL, United States; ^2^ Division of Interventional Radiology, Department of Radiology, Massachusetts General Hospital and Harvard Medical School, Boston, MA, United States; ^3^ Department of Microbiology and Immunology, College of Arts and Sciences, University of Miami, Coral Gables, FL, United States; ^4^ Department of Hospital Medicine, Flowers Hospital, Dothan, AL, United States

**Keywords:** colorectal metastases, hepatic colorectal metastases, interventional oncology, interventions in colorectal metastases, TARE, TACE, percutaneous ablation, DEBIRI-TACE

## Abstract

Colorectal malignancy is the third most common cancer and one of the prevalent causes of death globally. Around 20-25% of patients present with metastases at the time of diagnosis, and 50-60% of patients develop metastases in due course of the disease. Liver, followed by lung and lymph nodes, are the most common sites of colorectal cancer metastases. In such patients, the 5-year survival rate is approximately 19.2%. Although surgical resection is the primary mode of managing colorectal cancer metastases, only 10-25% of patients are competent for curative therapy. Hepatic insufficiency may be the aftermath of extensive surgical hepatectomy. Hence formal assessment of future liver remnant volume (FLR) is imperative prior to surgery to prevent hepatic failure. The evolution of minimally invasive interventional radiological techniques has enhanced the treatment algorithm of patients with colorectal cancer metastases. Studies have demonstrated that these techniques may address the limitations of curative resection, such as insufficient FLR, bi-lobar disease, and patients at higher risk for surgery. This review focuses on curative and palliative role through procedures including portal vein embolization, radioembolization, and ablation. Alongside, we deliberate various studies on conventional chemoembolization and chemoembolization with irinotecan-loaded drug-eluting beads. The radioembolization with Yttrium-90 microspheres has evolved as salvage therapy in surgically unresectable and chemo-resistant metastases.

## 1 Introduction

Colorectal cancer (CRC) is the third most prevalent malignancy in the United States and the third most common cause of death pertinent to cancer (1). The incidence of CRC has been increasing by approximately 3.2% per year and 2.5 million cases are estimated to be diagnosed in 2035 (2, 3). Around 56% of the patients lose their life from CRC (4). The mortality could be attributed to distant organ metastases noticed in 25% of patients at the time of initial diagnosis and in 50% of patients during disease progression (5). The 5-year survival rate of CRC confined to primary location is 88-91.1%, while the rate falls to 13.3-14% in metastatic CRC (6). Liver (68-75%) followed by lung (21-33%), distant lymph nodes (16-26%), bone (10.7-23.7%), peritoneum (11-15%), brain (0.3-0.6%), adrenal glands and spleen are the most to least common sites of CRC metastases (7, 8).

Synchronous colorectal cancer liver metastases (CRLM) are encountered in 20-25% of CRC patients whereas metachronous CRLM is observed in 20-30% of CRC patients (9, 10). Untreated CRLM has worse prognosis with a median survival of 4.5 to 12 months subject to the extent of disease at diagnosis (10). The intention of any curative treatment is to achieve the R0 resection of both the primary and metastatic tumor. Surgical resection is the potential curative and gold standard treatment for CRLM (11). It has improved the 5-year overall survival (OS) rate to 24-58% and a 10-year survival rate to 28% (10, 12–16). Although 50-60% of patients benefit from curative surgical resection of CRLM, only 10-25% of patients are suitable for surgery owing to tumor anatomy, extrahepatic involvement and general health status (10, 17, 18). Neoadjuvant systemic chemotherapy allows for sufficient tumor shrinkage for resection in merely 10-30% of non-surgical candidates (19). Current chemotherapy regimens include 5-fluorouracil and oxaliplatin (FOLFOX), 5-FU and irinotecan (FOLFIRI), and capecitabine and oxaliplatin (CapOx). These regimens have a response rate of 40% and an OS of 57% at 15-20 months (20). The addition of biologic agents to systemic chemotherapy such as anti-vascular endothelial and anti-epidermal growth factors inhibitors has improved the OS to >24 months (20). However, these systemic therapies are intolerable to a 1/3^rd^ of patients resulting in discontinuation of treatment. A few patients may experience chemotherapy-associated liver injury (CALI) including sinusoidal obstruction syndrome and steatohepatitis (20). Hence, the demand for locoregional therapies has increased to make the tumor amenable to resection in addition to mitigating unwanted side effects of chemotherapy. Minimally invasive interventional therapies such as percutaneous ablation, trans-arterial chemoembolization (TACE), trans-arterial radioembolization (TARE) and portal vein embolization (PVE) have transformed the management algorithms of CRLM. These therapies improve the candidacy for surgical resection, provide curative treatment options for non-surgical candidates, and improve the survival of patients undergoing palliative care (**Table 1**).

**Table 1:** Interventional Therapies for CRLM.

Indication	Treatment Options
Improve surgical candidacy	Portal vein embolization
	Lobar TARE
	Combine ablation with surgical resection
Therapies with Curative Intent	Ablation +/- Systemic chemotherapy
	Radiation Segmentectomy
	Firstline Chemotherapy plus TARE
Therapies with Palliate Intent	TARE
	TACE

CRLM, Colorectal liver metastases; TARE, Trans-arterial radioembolization; TACE, Trans-arterial chemoembolization.

## 2 Therapies to improve surgical candidacy

### 2.1 Portal vein embolization

**Figure 1:**
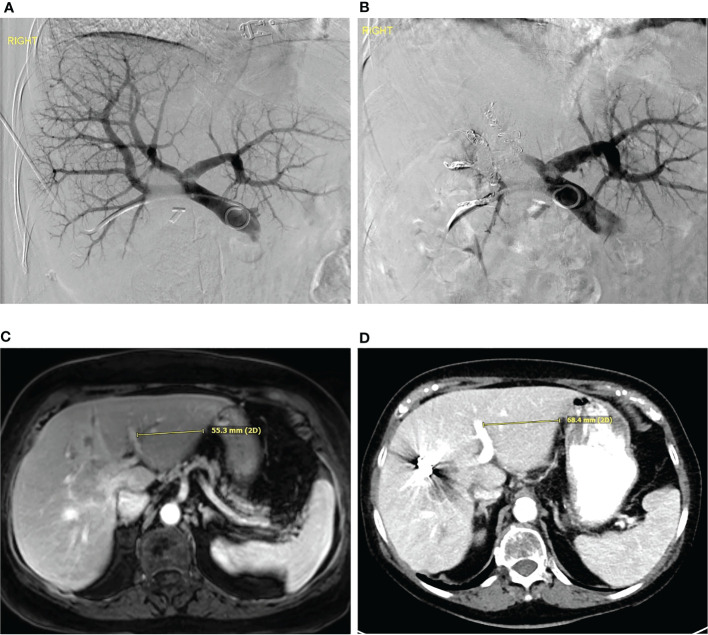
Portal vein embolization **(A)** Digitally subtracted percutaneous transhepatic portovenogram demonstrates patent main, left and right portal veins. The portal vein was embolized with particles followed by coil embolization. **(B)** Digitally subtracted portovenogram after portal vein embolization demonstrates flow only to the left hepatic lobe. **(C)** Pre-procedure MRI and **(D)** post portal vein embolization CT demonstrates hypertrophy of the left hepatic lobe.

One of the main limitations of curative surgical resection is the presence of low volume of the future liver remnant (FLR), which might lead to hepatic insufficiency following the surgery (21). In the last few decades, various techniques have been introduced to induce hypertrophy of the FLR, thereby preventing the liver failure. In 1980s, Masatoshi Makuuchi introduced PVE of right portal vein to cause hypertrophy of the left lobe of the liver (22). PVE diverts blood flow to the healthy liver through embolization of the portal vein branches of the diseased liver. This results in atrophy of the embolized liver and hypertrophy of the non-embolized liver (**Figure 1**). The resultant increased FLR makes it possible to resect the large or multiple liver tumors. The exact mechanism of liver atrophy-hypertrophy following PVE remains unclear. However, it is hypothesized to be due to (i) upregulated cytokines and growth factors during liver regeneration, (ii) restituted increase in hepatic arterial perfusion and (iii) cellular host response enhancing the local tumor growth (23).

PVE has become the standard of practice for patients with inadequate FLR prior to extensive hepatic resection. The FLR of <20% in the normal liver, <30% in the liver with chemotherapeutic exposure, and <40% in the cirrhotic liver is usually considered an indication of PVE (11, 24–27). The liver regenerates by 20-46% in 6-8 weeks following the procedure (28). The resection rate after PVE is reported to be around 60-80%, 20% of patients may present with insufficient FLR hypertrophy or tumor progression (29–31). Other complications include tumor recurrence and accelerated tumor growth following the procedure (11). Tumor progression is the major concern as it affects the clinical and survival outcomes and may lead to unresectable disease. Pamecha et al. reported increased tumor growth rate among post-PVE cases compared to controls (0.36± 0.68 ml/day vs. 0.05± 0.25 ml/day; P=0.06) (**Table 2**) (29). For patients with high tumor load, defined as ≥ 9 CRLM or ≥ 5.5 cm diameter for the largest metastatic lesion, a liver transplant may be the preferred management for improved survival (32). Dueland et al. reported a 5-year survival rates of 33.4% and 6.7% in patients who underwent liver transplant and post-PVE liver resection respectively (32).

**Table 2:** Data on PVE for CRLM.

Study	Study design	Country/region	Sample size	Treatment	Follow up time/Inclusion period	Outcome
Dueland et al., 2021 (32)	Retrospective study	Norway	53	PVE prior to liver resection compared with liver transplantation	Included the patients between 2006-2019	5-year OS for patients with PVE + Liver resection: 44.6%; 5-year OS for HTL patients was 33.4% and 6.7% in liver transplant and PVE groups respectively; 5-year OS rate for patients with HTL+ left-sided primary tumors was 45.3% and 12.5% in liver transplant and PVE groups respectively. Median OS from the PVE and liver resection was 32.7 months
Huiskens et al., 2017 (33)	Retrospective study	Netherlands	Cases: 46 PVE patients who underwent liver resection; controls: 46 non-PVE patients who underwent liver resection	PVE followed by liver resection vs. liver resection alone	Included the patients between 2000-2015	No significant difference in 3-year DFS (16% vs. 9%; P=0.776) and 5-year OS (14% vs. 14%; P= 0.866)
Ironside et al., 2017 (34)	Systematic review	–	1345	Liver resection in PVE vs. non-PVE patients	Included the studies until 2016	Post-operative morbidity: 42% in PVE and 35% in non-PVE patients; Median OS in PVE and non-PVE patients following resection was 38.9 months and 45.6 months respectively; Median DFS in PVE and non-PVE patients was 15.7 months and 21.4 months respectively.
Giglio et al., 2015 (35)	Meta-analysis	–	688	Liver resection in PVE vs. non-PVE patients	Included the studies until 2015	No significant difference was observed between PVE and non-PVE groups in tumor recurrence (OR: 0.78; 95% CI: 0.42-1.44), 3-year OS (OR: 0.80; 95% CI: 0.56-1.14) and 5-year OS (OR: 1.12; 95% CI: 0.40-3.11)
Hoekstra et al., 2012 (36)	Retrospective study	United States	28	Liver resection in PVE vs. non-PVE patients	Included the patients between 2004-2011; After liver resection, median follow up of 6 months in PVE group and 40 months in non-PVE group.	25% of patients developed new lesions in FLR and 42% had tumor recurrence post PVE; 11% of the tumors were not resectable post PVE. 3-yr OS was 77% vs. 26% in non-PVE vs. PVE groups respectively.
Simoneau et al., 2012 (37)	Prospective study	Montreal, Quebec	109 cases and 11 controls	Liver regeneration in PVE vs. non-PVE group	Included the patients between 2003-2011	33.4% increase in TV in right lobe and 49.9% increase in TV in left lobe post-PVE; Growth rate: no statistical significance; Median FLR was similar in test group and control (28.8% vs. 28.7%)
Pamecha et al., 2009 (29)	Prospective study	United Kingdom	22	Liver growth rate after PVE vs. non-PVE; All patients had chemotherapy (5FU, folinic acid, oxaliplatin/irinotecan) before and after PVE.	Included patients between 1999 to 2005.	Tumor volume at resection (P=0.98), time from presentation to resection and tumor growth rate after PVE (P=0.06), (P=0.19) were not statistically significant among PVE group compared to controls. Ki67 proliferation index (P= 0.048) was significantly higher than in controls. The 5-year survival rate in PVE vs control group: 25% vs. 55%; The median DFS in PVE vs control groups: 12 months vs. 24 months.
Pamecha et al., 2009 (38)	Retrospective study	United Kingdom	101	Cases: 36 patients underwent preoperative PVE Controls: 65 patients	Included patients between 1999 to 2005	The median volume of FLR increased from 22% to 32% following PVE; Overall morbidity in cases and controls was 36% and 20% respectively; 1-, 3- and 5-year survival following PVE was 70%, 30% and 25% respectively; 3- and 5-year survival after liver resection in cases vs. controls was 52% vs. 65% and 25% vs. 50% respectively. No significant difference in recurrence rates between cases and controls.
Mueller et al., 2008 (24)	Retrospective study	Germany	107	Outcomes after liver resection in PVE vs. non-PVE patients	Included patients between 1995 to 2004	81% of patients were unresectable due to tumor progression post PVE; Progressive metastases: 52.4%; 5-year survival rate: 43.66%
Kokudo et al., 2003 (39)	Retrospective study	Japan	47	Cases: 18 patients who underwent pre-operative PVE Controls: 29 patients without PVE	Included patients between 1996 to 2000	Tumor volume increased by 20.8% and percent tumor volume increased by 18.5% post PVE; OS in PVE group: 59.7% and 47.8% at 2 and 4 years respectively; whereas in control group: 67.8% and 50.2% at 2 and 4 years respectively (P= 0.421); DFS in PVE group: 15.2% and 0% at 2 and 4 years respectively; in control group: 45.8% and 34.4% at 2 and 4 years respectively.

PVE, Portal vein embolization; CRLM, Colorectal liver metastases; OS, Overall survival; HTL, High tumor load; DFS, Disease free survival; FLR, Future liver remnant; TV, Tumor volume; 5FU, 5-Fluorouracil.

### 2.2 Lobar trans-arterial radioembolization

The external beam radiotherapy (EBRT) of the liver exposes the normal hepatic parenchyma to radiation, in addition to the target tumor tissue. Even 35-45Gy, a dose inadequate to induce tumor cell death, can cause radiation-induced liver disease in 50% of the patients due to the low radiation toxicity threshold of normal hepatic parenchyma (40, 41). TARE, also known as selective internal radiation therapy (SIRT) deploys microspheres made of glass or resin and loaded with Yttrium-90 (Y-90) into the hepatic tumor vasculature. The Y-90 TARE emits beta radiation to the selective tumor tissue in contrast to the whole hepatic parenchyma in EBRT. As the radiation is achieved through the infusion of Y-90 microspheres into the hepatic artery, the TARE technique is often referred to as “inside-out radiation” or brachytherapy (42). The Y-90 TARE delivers the radiation with a mean penetration of 2.5 mm, mean energy of 0.94 MeV and targeted radiation dose of 80-150 Gy to the tumors (43).

The concept of lobar TARE as a method to increase the FLR while also controlling the tumor growth in the diseased liver is recently popularized (**Table 3**). Teo et al. studied seven retrospective clinical studies involving the patients undergoing lobar TARE and reported a FLR hypertrophy of 26-47% within 1.5-9 months of the procedure (47). However, Nebelung et al. reported a significantly greater hypertrophy in patients after PVE than lobar TARE (25.3% vs. 7.4%; P<0.001) (45). However, the post-TARE hypertrophy was substantial with a minimized risk of tumor progression in the embolized lobe (48). Edeline et al. stated that the increase in FLR was similar after TARE as well as PVE procedures (49). Kurilova et al. reported two cases reports in which the patients had insufficient FLR post PVE and underwent lobar TARE. They observed 13% increase in FLR at 4-week follow up in the first patient and 59% increase in FLR at 7-week follow-up in the second patient (50). Liebl et al. studied the FLR hypertrophy in pigs and reported that although PVE induced rapid FLR hypertrophy, it reached a plateau after I month of procedure, whereas, TARE resulted in FLR comparable to PVE within 3-6 months of procedure (51). Vouche et al. studied 83 patients with unilobar disease and observed a reduction in the tumor volume from 134 cc to 56 cc during >9 month follow up period (46). Another study by Edeline et al., including 34 patients, delivered a median lobar dose of 122 Gy and observed a complete response rate of 0%, partial response rate of 26%, stable disease in 63%, and progression of disease in 3% of patients based on RECIST criteria (49). However, CR, PR, SD and PD of 30, 33, 30 and 2% were reported based on mRECIST criteria. Edeline et al. also reported a median OS of 13.5 months and a median time to tumor progression of 21.7 months (49). The lobar TARE has the advantage of tumor control and biological test of time for extrahepatic tumor progression prior to liver resection. Lobar TARE is a well-tolerated procedure with very minimal side effects such as pain and nausea. A few studies reported an increase in Child-Pugh score from 6 to 7 during the first 6 months follow-up which improved later during the >6-9 month follow up period (52). A > 20% increase in the splenic volume was reported without any signs of hypersplenism or additional findings of portal hypertension (52). Serious toxicities including irreversible ascites, temporary and progressive hyperbilirubinemia, and variceal hemorrhage may be observed following the procedure (49).

**Table 3:** Data on lobar TARE in CRLM.

Study	Study design	Country/region	Sample size	Treatment	Follow up time/Inclusion period	Results
Chiu et al. (44) 2023	Retrospective study	United States	16	Radiation segmentectomy with Y90 in oligometastatic disease (well-controlled primary tumor, ≤ 3 metastases, absence of active extrahepatic tumor burden.	Included patients between 2009 and 2020	Disease control rate was 93%; 13.3% achieved complete response and 47% had partial response. 40% of the patients required subsequent systemic or local tumor therapy while 60% underwent additional chemotherapy. Median time-to-progression was 72.9 months.
Nebelung et al., 2021 (45)	Retrospective study	Germany	73	Lobar TARE: 24 patients; PVE: 49 patients	Included patients who underwent PVE between 2015 to 2019 and TARE between 2013 to 2019	Hypertrophy after PVE was significantly greater than that after TARE (25.3% vs. 7.4%; P<0.001); When stratified by the presence of cirrhosis, the difference in hypertrophy was statistically significant in those without cirrhosis but not statistically significant in cirrhotic patients.
Vouche et al., 2013 (46)	Retrospective study	United States	83	83 patients with uni-lobar disease treated with Y90 microspheres; HCC: 67 patients; CRLM: 8 patients (6 patients had ≥1 cycle of chemotherapy); Cholangiocarcinoma: 8 patients	Included patients between 2003 to 2012	FLR hypertrophy increased from 7% at one month to 45% at 9 month follow up; Median FLR hypertrophy: 26%; Reduction in tumor volume was observed from 134 cc to 99 cc at 3-month period and to 56 cc at > 9-month period
Teo et al., 2016 (47)	Systematic review	Singapore	312	312 patients (HCC: 215 patients; intrahepatic cholangiocarcinoma: 12 patients; CRLM: 85 patients)	Included studies between 2000 to 2014	FLR hypertrophy ranged from 26-47% over a period of 44 days to 9 months
Garlipp et al., 2013 (48)	Retrospective study	Germany	176	Lobar TARE: 35 patients; PVE: 141 patients	Included patients between 2006 and 2012	FLR hypertrophy was significantly greater in PVE group than TARE group (61.5% vs. 29%; P<0.001)

TARE, Trans-arterial radioembolization; PVE, Portal vein embolization; HCC, Hepatocellular carcinoma; CRLM, Colorectal liver metastases; FLR, Future liver remnant.

### 2.3 Combined RFA and surgical resection

A few studies recommend the combination therapy of RFA with surgical resection to slightly improve the survival and recurrence risk compared to RFA alone (**Table 4**). Mima et al. studied the efficacy of RFA alone and RFA combined with hepatic resection in unresectable CRLM (53). RFA was mainly performed alongside hepatic resection in those patients who had an effective clinical response to preoperative chemotherapy (FOLFOX). Metastatic nodules smaller than 2 cm was the main indication for RFA while the contralateral tumor was for the hepatic resection. The 3-year recurrence free survival was 33.2% in hepatic resection alone group and 18.5% in combined hepatic resection+ RFA group. Although tumor recurrence was reported in both the group of patients, it was not statistically significant (P=0.154). The 3-year PFS was 45.3% in hepatic resection alone group compared to 12.8% in hepatic resection + RFA group (P= 0.472). The 3- and 5-year OS was 70.4% and 62.6% in hepatic resection group and 77.1% and 64.3% in the hepatic resection + RFA group (P= 0.627) (53). Mima et al. concluded that hepatic resection combined with RFA may be a safe and effective alternative after responsive chemotherapy (53) The similar conclusion was observed in a retrospective study by Sasaki et al. (54). They observed improved resection rates in the resection +RFA group compared to resection alone group (15.1% vs. 8.5%; P= 0.071) (54).

**Table 4:** Data on combined percutaneous ablation and surgical resection.

Study	Study design	Country/Region	Sample size	Treatment	Follow up/Inclusion period	Results
Mima et al., 2013 (53)	Prospective study	Japan	153	118 patients with unresectable CRLM treated preoperatively with FOLFOX ± bevacizumab; HR alone: 35 patients; HR + RFA: 13 patients	Included patients between 2005 to 2010	Postoperative morbidity: 17% in HR group and 23% in HR+RFA group (P= 0.640); Local tumor recurrence at RFA site in only one tumor (7.7% of patients); 3-year PFS: 45.3% in HR group and 12.8% in HR+RFA group (P= 0.472); 3-year OS rate: 70.4% in HR group and 77.1% in HR+RFA group (P=0.627)
Sasaki et al., 2016 (54)	Retrospective study	United States	485	Resection + RFA: 86 patients; Resection alone: 399 patients	Included patients between 2003 to 2015	R1 resection was more frequent in surgical resection + RFA group than the resection-alone group (15.1% vs. 8.5%; P= 0.071); Median OS for combined and resection alone groups: 20.7-61.8 months and 75.3 months respectively; 5-year OS for combined and resection alone groups: 52.7% and 58.7% respectively.

CRLM, Colorectal liver metastases; FOLFOX, 5-fluorouracil and oxaliplatin; HR, Hepatic resection; RFA, Radiofrequency ablation; PFS, Progression free survival; OS, Overall Survival.

## 3 Therapies with curative intent

### 3.1 Ablation +/- systemic chemotherapy

Percutaneous thermal ablation is a tumor-destructive technique and is based on exposing the tumor cells to a targeted temperature of > 60^0^ C or < -40^0^ C. Ablation can be accomplished through thermal techniques such as radiofrequency, microwave, cryoablation, laser ablation, and focused ultrasound ablation. The irreversible electroporation (IRE), a nonthermal ablation technique utilizes an electrical field to induce tumor death without damaging the tissue protein architecture of vessels and the bile-ducts (55). Either thermal or non-thermal, ablation techniques have the advantages of being minimally invasive and less morbid than surgical resection and can be delivered as an out-patient treatment. The open or percutaneous approach to thermal ablation has been studied in the literature. Puijk et al., reported significantly improving liver tumor PFS following percutaneous ablation (2010-2013: 37.7%; 2014-2017: 69%; 2018-2021: 86.3%; P< 0.0001) whereas the PFS was stable following open ablations (2010-2013: 87.1%; 2014-2017: 92.7%; 2018-2021: 90.2%; P= 0.12) (56). The complications were less severe in percutaneous rather than open approach (2010-2013: P=0.69; 2014-2017: P= 0.129; 2018-2021: P= 0.02) (56). The tissue damage secondary to ablation is low when compared to surgical resection, which is the most important requisite in patients with underlying liver disease or those who already had extensive liver resection (55).

RFA is a well-studied and most widely used ablative modality in colorectal metastases. The monopolar or bipolar radiofrequency ablation (RFA) systems produce ionic oscillation by a high-frequency alternating current resulting in frictional heating and tissue damage (57). The level of thermal tissue damage varies depending on the achieved temperature. For instance, a 50-55^0^ C for a period of 4-6 minutes induces irreversible cellular damage, 60-100^0^ C leads to irreversible coagulation of the cells and 100-110^0^ C results in vaporization and carbonization of tissue (58). The 1, 3, 5,10-year survival rates of CRLM following RFA are 98%, 69%, 48%, and 18% in a study by Solbiati et al. (59). Local tumor progression (LTP) after RFA, seen in 2-60% of cases, is an important factor to consider while ablating the CRLM. There are many factors that attribute to LTP including tumor size, tumor number, ablation zonal geometry, ablative margin, extrahepatic disease, location adjacent to large vessels and subcapsular tumors (60, 61). Radiofrequency ablation (RFA) is usually recommended in patients with ≤ 3-5 metastases of size ≤ 3-3.5 cm, not involving bile ducts or large vessels (≥3 mm), and not located centrally (62, 63).

Tumor size is critical in selecting patients for RFA as the commercially available devices can deliver the ablation to about 4-5 cm in one session and the studies reported high success rates of RFA in tumors ≤ 3-4 cm. In a study by Nielsen et al., the local recurrence after ablation was reported in 9%, 26.5%, and 45% of metastases measuring 0-3 cm, 3-5 cm and > 5 cm respectively (64). Compared to surgical resection, RFA has a lower complication rate (9.5%) and minimal risk of death (10, 65). However, it cannot replace surgical resection, especially in tumors > 3 cm size (57). The number of CRLM is also an important criterion when selecting the patients for RFA. Solitary CRLM is associated with high tumor control and survival rates. Kim et al. reported the 5-year survival and disease-free survival rates as 51% and 34% respectively in patients with solitary CRLM of size < 3 cm (66). Similarly, Gillams et al. studied the 5-year survival rate of solitary CRLM of size 2.3 cm to be 54% with a median survival of 63 months (67). Wang et al. studied the emphasis of ablative and tumor margins and reported that the risk of LTP decreases by 46% for every 5-mm increase in ablative margin size and increases by 22% with every 5 mm increase in tumor size (68). The tumor abutting large vessels causes convective heat loss termed as “heat-sink effect”, hence preventing heat accumulation in the tumor (63). A study by Elias et al. reported that 23% of CRLM, close to the large vessels, recured compared to 3% of CRLM located away from the vessels (69). In such situations, percutaneous balloon occlusion of large vessels during RFA has demonstrated improved tumor progression rates (62). Van Tilborg et al. studied that the centrally located CRLM recur more often compared to peripheral CRLM (21.4% vs. 6.5%; P= 0.009) (10).

Local tumor progression following RFA can be re-treated with repeat RFA, stereotactic body radiation therapy (SBRT), TACE, hepatic resection, and ultimately transplantation; however, with a high failure risk (70). The optimal choice among these techniques is still debatable, and a study by Xie et al. compared the repeat RFA with TACE and transplantation (70). In their study, repeat RFA has comparable outcomes with transplantation; hence the former is the primary choice, while the latter can be performed in patients where RFA failed or is inapplicable (70). Recently, CT-guided I^125^ brachytherapy has been studied in patients with recurrent HCC after thermal ablation. Its validation in recurrent CRLM is yet to be determined.

**Figure 2:**
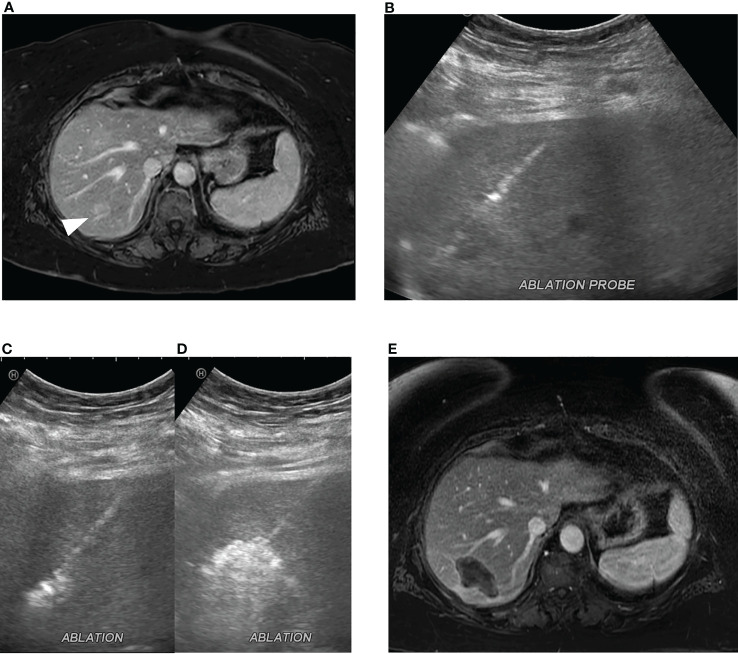
Microwave ablation of colorectal cancer liver metastasis. **(A)** A 2.0 cm colorectal cancer metastasis in segment 7 (white arrowhead). **(B)** Ultrasound-guided microwave ablation probe placement in the segment 7 lesion which was confirmed with CT (not shown). Continuous monitoring of ablation was performed with ultrasound with **(C)** early and **(D)** late ablation images obtained. **(E)** Post-ablation MRI, 1 month post procedure, demonstrates ablation zone without evidence of residual disease.

Other ablation techniques include microwave ablation (MWA), irreversible electroporation (IRE), and cryoablation. MWA has shown to be effective as an alternative to RFA and in a few cases, it is the preferred modality. The MWA generates heat by utilizing electromagnetic signals. Current machines operate between 900-2450 MHz, a frequency at which the microwaves cause coagulation necrosis by the oscillation of polarized water molecules which produce friction and heat (**Figure 2**) (57, 71). Compared to RFA, the size and zone of MWA are consistent and less affected by the heat-sink effect, impedance, penetrability, and thermal conductivity (72, 73). Gravante et al. examined the histopathological sample of MWA tissue and found no viable cells 6 cm away from the ablation zone in 93% of cases (74). Ierardi et al. included patients who are unfeasible to RFA such as those with tumors > 3 cm and are abutting larger vessels (> 3 mm) (73). They reported that the local recurrence was observed in 13% of patients with a disease-free OS of 20.5 months. Although no major complications were noticed, approximately 45% of patients had minor complications such as abdominal pain, fever with malaise, nausea, vomiting and elevated serum bilirubin levels (73). Pathak et al. reviewed various studies on RFA and MWA and reported that the local recurrence rates of CRLM after RFA and MWA to be ranging from 10-36% and 5-13% respectively (71). IRE is a non-thermal ablative technique that induces high-voltage electrical pulse waves between the electrodes (75). It is a safer ablation method in case of tumors close to the vascular or biliary structures due to the absence of the heat-sink effect (76, 77). The COLDFIRE-1 is a Phase-I study that demonstrated CRLM death and necrosis when exposed to IRE (78). COLDFIRE-2 is a Phase-II study including the patients with ≤ 5 cm CRLM, and it reported a 1-year PFS of 68%. Around 74% of the patients achieved local tumor control after the repeat IRE procedure (79). In a study by Schicho et al., 67% of patients achieved tumor control after the first IRE and 96% after re-intervention (80). Complications during IRE were reported to be observed in 40% of patients, with the most severe being arrhythmias, portal vein thrombosis, and biliary obstruction (79).

Laser ablation uses micrometer optical fiber to produce heat by transmitting infrared light. The optical fiber is connected to a generator or diode made of neodymium: yttrium aluminum garnet (ND: YAG), which emits a precise wavelength. The size of the fiber, the wavelength used, conduction and penetration of surrounding tissue, and the power and duration of the ablation are the factors that affect the size of the ablation zone (81). The lesions located within 1 cm of the main biliary duct, untreatable coagulopathy, and ascites interposed along the path of the applicator are considered contraindications to the thermal ablation (82). Patients may experience side effects after the procedure including pain, and post-ablation syndrome. Pain is self-limiting and depends on the size of the ablation zone. Post-ablation syndrome presents with flu-like illness with low-grade fever, nausea, vomiting and malaise, and can be managed symptomatically (72). Complications of the ablation procedure can be secondary to the injury to surrounding structures or the ablation itself, such as pneumothorax, intraperitoneal bleeding, hemothorax, portal vein thrombosis, gastrointestinal tract perforation, strictures, bile duct injury, cholecystitis, and liver abscess (72, 82).

The EORTC-CLOCC trial was a phase-II clinical trial that studied the efficacy of systemic chemotherapy with or without RFA in 119 patients diagnosed with unresectable CRLM (83). The trial randomized patients to receive systemic treatment alone or in combination with RFA. A significant improvement in OS and PFS was reported in the combined modality group rather than the systemic chemotherapy alone group (83). Improved OS in the combined modality group compared to the systemic treatment alone group (HR: 0.58; P=0.01) was observed. The 3-, 5- and 8-year OS rates were 56.9%, 43.1%, and 35.9% respectively in the combined modality group, and 55.2%, 30.3%, and 8.9% respectively in the systemic chemotherapy alone group. The median OS was 45.6 months in the combined modality group and 40.5 months in the systemic chemotherapy alone group. There was a prolonged PFS in the combined modality group (HR: 0.57; P=0.05). The liver as the first site of recurrence was noticed in 46.7% of the combined modality group and 78% of the systemic chemotherapy alone group (83). Another study, the ARF2003, included 52 unresectable CRLM treated with neoadjuvant chemotherapy and RFA. The study reported complete hepatic response in 75% of patients at their 3-month follow-up. The OS at 1-, and 5-years was 94% and 43% respectively, whereas PFS was 46% and 19% (84). Furthermore, another study reported that the combination of RFA and systemic chemotherapy has shown improved 3-year progression-free survival in comparison to systemic chemotherapy alone (27.6% vs. 10.6%; hazard ratio= 0.63; CI: 0.42-0.95; P=0.025) (85).

SBRT delivers the radiation to a specified region of interest with millimetric accuracy and reduces the irradiation to surrounding parenchyma. Unlike RFA and MWA, the SBRT is the better technique to access the perihilar, periampullary, or subcapsular lesions (86). SBRT can be considered, in combination with surgical resection, for oligometastatic liver disease that failed local therapies (87). Candidates with ≤ 5 CRLM involving <700 cc of the liver, an expected survival of > 3 months, curative extrahepatic disease, no chemotherapy received before two weeks of planned SBRT, and ≤ 2 Eastern Cooperative Oncology Group performance status are suitable for SBRT (87). A radiotherapy dose of ≥ 100-110 Gy can achieve local tumor control in 80-90% of the patients, while a higher dose may be required in case of larger tumors to attain similar outcomes (86–88). A study by Petrelli et al. included 656 patients and reported that the SBRT provides an overall survival of 67% and 57% and local tumor control of 67% and 59% at 1 and 2 years, respectively (89, 90). Compared to RFA, SBRT achieves greater 2-year local tumor control (84% vs. 60%); however, both the techniques had similar OS rates (91). The OLIVER trial compares the SBRT and chemotherapy alone and may provide further validations for its application (NCT03296839).

### 3.2 Radiation segmentectomy

Radiation segmentectomy (RS) delivers a very high ablative radiation dose (>190 Gray) confined to one or two liver segments, thus limiting the radiation-related complications (92, 93). The dose is based on the available literature for RS in patients with HCC which demonstrated a correlation between the level of tumor necrosis and the radiation exposure (93). The major intent of RS is to achieve cure in patients with CRLM, similar to the ablation or ablative external radiation therapy (94, 95). Diagnostic and therapeutic advancements through proper patient selection, imaging and radiation dosimetry allowed transition of lobar salvage to segmental curative radioembolization, especially in patients with features including (i) solitary tumor of size ≤ 5 cm (ii) primary or secondary liver tumor without other organ involvement and (iii) a tumor that can be targeted angiographically such that ≤ 2 liver segments receive the ablative dose of radiation (92, 96).

RECIST criteria have been widely employed to evaluate the response to TARE, however, PRECIST has proved to be more accurate in CRLM (97–99). Among all the parameters included in PRECIST, metabolic tumor volume and total lesional glycolysis are observed to be the significant predictors of OS (100). Recently, Choi criteria based on tumoral attenuation and diameter on CT imaging was identified to be a reliable criterion in CRLM to predict the PFS (101). Kurilova et al. observed that the RS of ≤ 3 hepatic segments can provide a 2-year tumor control rate of 83% in patients with limited therapeutic options and limited metastatic disease (**Table 5**) (102). They also reported that the tumor progression occurred in 21% of their study population which is similar to the study by Padia et al. (103) who reported tumor progression in 28% (102). In a study by Meiers et al, the authors included 10 patients of which 7 patients had inoperable CRLM confined to ≤ 2 liver segments (104). The procedure was unsuccessful in one among 7 patients due to attenuated hepatic vasculature. Of the remaining 6 patients with CRLM, four had a complete response or stable disease at their follow-up evaluation ranging from 1-14 months. Two of six patients had progressive disease after 7- and 18-months period. There were no reported adverse events. The mean PFS was 7.1 months for the entire cohort (92, 104).

**Table 5:** Studies describing the efficacy of radiation segmentectomy.

Study	Study design	Country/Region	Sample size	Patient characteristics	Follow-up/Inclusion period	Results
Kurilova et al., 2021 (102)	Retrospective study	United States	10 patients	14 tumors treated with 12 RS sessions; Each patient has ≤ 3 tumors of median size 3 cm; Median radiation dose delivered: 293 Gy	Included the patients between 2015 and 2017; median follow up of 17.8 months (Range: 1.6-37.3)	Tumor response as per Choi and RECIST criteria: 100% and 44% respectively; Tumor progression: 33%; 1-, 2- and 3-year PFS: 83%, 83%, and 69% respectively; Median OS: 41.5 months
Padia et al., 2020 (103)	Retrospective study	United States	36	36 patients; 81% had prior chemotherapy; CRC: 31%; NEN: 28%; Sarcoma: 19%; Miscellaneous: 22%	Included patients between 2013 and 2018	Disease control rate was 92% according to RECIST criteria in all tumors and 100% according to mRECIST criteria in hypervascular tumors; Tumor progression: 28%; OS at 6 and 12 months was 96% and 83% respectively.
Jia et al., 2019 (96)	Systematic review	Multiregional	155	HCC: 145; CRC: 7; Others: 3	Included patients between 1991 and 2018	CR, PR, SD and PD was observed in 20-82%, 10-70%, 1.8-40% and 0-8% respectively. Disease control rate: 92-100%.

PFS, Progression free survival; OS, Overall survival; CRC, Colorectal cancer; NEN, Neuroendocrine neoplasm; HCC, hepatocellular carcinoma; CR, Complete response PR, Partial response; SD, Stable disease; PD, Progression of disease.

Although RS has a promising role in the treatment of HCC that cannot be resected or ablated, the literature on CRLM is limited (93, 105–107). In addition, as the most of the CRLM patients may have been pre-treated with chemotherapeutic regimens, the hepatic vasculature can be altered limiting the ability to perform the super-selective RS. Furthermore, the hypovascular nature of CRLM results in difficulty targeting the tumor. Based on the available data, RS appears to provide local tumor control with acceptable toxicity in patients with CRLM. Further studies on patient selection and tumor response are required to emphasize the application of RS in patients with CRLM.

### 3.3 Firstline chemotherapy plus TARE

Combined therapy with radioembolization and systemic chemotherapy has been studied in the literature. Haber et al. reported 38-month and 25-month median survival of CRLM patients treated with combined systemic chemotherapy plus TARE and systemic chemotherapy alone groups, respectively from the date of primary diagnosis (108). Three phase-III clinical trials, SIRFLOX, FOXFIRE and FOXFIRE-Global, studied the efficacy of combined chemotherapy with Y90 TARE over chemotherapy alone among 1103 patients in total (109–111). SIRFLOX trial by Van Hazel et al. concluded that the addition of TARE to the chemotherapy did not improve the PFS, however delayed the tumor progression significantly (**Table 6**) (110). A combined analysis of FOXFIRE, SIRFLOX and FOXFIRE-Global was performed by Wasan et al. with a total of 1103 patients (113). The patients were randomized to receive FOLFOX alone (549) or in combination with single cycle of TARE (554). Higher overall response rate was reported in the combined group (72% vs. 63%) however no differences were identified in median OS (22.6 months vs. 23.3 months; P=0.61). Radiological progression of the tumor was observed in 49% of FOLFOX alone group and 31% of the combined group. The cumulative incidence of tumor progression in the first 12 months follow up period was 22% in the combined group compared to 39% in FOLFOX alone group. An objective response rate was reported in 72% of the combined group and 63% of FOLFOX alone group (P= 0.0012). The study also reported high odds of grade 3 or worse adverse events in the combined group (74%) than the FOLFOX alone group (67%) (OR: 1.42; P= 0.008) (113). Wasan et al. reported 17% resectablity rate in TARE + chemotherapy group and 16% in chemotherapy alone group (P=0.67) (113). Garlipp et al. reported an improved resectability rate of the lesions after TARE+ chemotherapy compared to chemotherapy alone (38.1% vs. 28.9%; P<0.001) (115). The subgroup analyses of the FOXFIRE, SIRFLOX and FOXFIRE-Global trials reported no significant difference in OS between the combined and FOLFOX alone group (112, 114). However, when tumors are stratified based on location, the addition of SIRT improved the OS in right-sided but not left-sided primary CRC (**Table 6**) (112, 114).

**Table 6:** Data on the efficacy of combined chemotherapy and TARE.

Study	Study design	Country/Region	Sample size	Patient characteristics	Follow up/Inclusion period	Results
Gibbs et al., 2018 (112)	Combined analysis of two randomized control trials FOLFOX and SIRFLOX	Multiregional study	739	FOLFOX + SIRT: 372 patients; FOLFOX alone: 367 patients	Included patients from 2006 to 2015; median follow-up period was 22.2 months	TARE has significant impact on OS in patients with right-sided (22 months vs. 17.1 months; P= 0.008) but not left-sided primary tumor (24.6 months vs. 26.6 months; P= 0.264).
Wasan et al., 2017 (113)	Combined analysis of three trials FOXFIRE, SIRFLOX, FOXFIRE-Global	Multiregional study	1103	FOLFOX+ SIRT: 554 patients; FOLFOX: 549 patients	Included patients between 2006 and 2014; Median follow-up was 43.3 months	No difference in median OS and median PFS; ORR: 72% (FOLFOX+SIRT) and 63% (FOLFOX alone); Tumor progression: 31% (FOLFOX+SIRT) and 49% (FOLFOX alone)
Van Hazel et al., 2017 (114)	Combined analysis of FOXFIRE-Global and SIRFLOX trials	Multiregional study	739	739 patients were randomized to receive either FOLFOX alone or in combination with SIRT with Y-90 microspheres	–	SIRT improved OS in right sided primary tumors (22 vs. 17 months; P= 0.007) and the difference in OS was not significant in left-sided primary tumors (24.6 vs. 25.6 months; P= 0.279)
Van Hazel et al., 2016 (110)	Randomized Phase III trial	Multiregional study	530	530 patients randomized to FOLFOX + SIRT +/- bevacizumab or FOLFOX	Included patients between 2006 and 2013;	Median PFS at any site: 10.2 (FOLFOX alone) vs. 10.7 (FOLFOX+SIRT) months (P= 0.43); Median PFS in the liver: 12.6 (FOLFOX alone) vs. 20.5 (FOLFOX+SIRT) months (P= 0.002); ORR at any site: 68.1% (FOLFOX alone) vs. 76.4% (FOLFOX+SIRT) (P= 0.113); ORR in the liver: 68.8% (FOLFOX alone) vs. 78.7% (FOLFOX+SIRT) (P= 0.042); Grade ≥ 2 adverse events observed in 73.4% (FOLFOX alone) and 85.4% (FOLFOX+SIRT) of patients.

FOLFOX, 5-fluorouracil and oxaliplatin; SIRT, Selective internal radiation therapy; TARE, Trans-arterial radioembolization; OS, Overall survival; ORR, Objective response rate; PFS, Progression free survival.

## 4 Therapies with palliative intent

### 4.1 TACE

Approximately 80% of blood supply to CRLM is derived from the hepatic artery while it is from the portal vein to the normal liver parenchyma (42, 116). Transarterial therapies utilize the advantage of dual blood supply of the liver and hence the cytotoxic agents infused through the hepatic artery selectively target tumor over normal cells. In addition, the first pass metabolism of the chemotherapeutic agents can be bypassed in the intra-arterial therapies. TACE is a catheter-based infusion of one or more chemotherapeutic medications and embolizing material into the hepatic artery. Embolizing material can be either temporary or permanent. The former includes collagen, gelatin sponge and degradable starch microspheres, while the latter include polyvinyl alcohol particles. Lipiodol has both the vaso-occlusive effect and the ability to enhance the effect of cytotoxic agents (117). TACE procedure was first introduced by Yamada et al. in late 1970s (118). In general, TACE is indicated as a second-line modality of treatment in patients who are refractory to systemic chemotherapy or in inoperable CRLM (119). Conventional TACE (cTACE) represents the injection of lipiodol + chemotherapy and embolizing agents. Recently, the drug-eluting beads are being used as embolic materials termed as DEB-TACE. The efficacy of cTACE and DEB-TACE have been extensively studied in the management of CRLM.

#### 4.1.1 Conventional TACE

The chemotherapeutic regimen and embolic materials are variable in the published studies. Albert et al. studied the efficacy of TACE with doxorubicin, cisplatin, mitomycin C and lipiodol mixture followed by embolization material- polyvinyl alcohol particles, in 245 unresectable CRLM in 121 patients who were refractory to systemic chemotherapy (120). Median survival from initial CRLM diagnosis and TACE was 27 months and 9 months, respectively. The study described that the OS was better with TACE after first- or second-line systemic chemotherapy than after three to five lines of systemic chemotherapy (11-12 months vs. 6 months; P= 0.03) (120). Vogl et al. studied 463 patients with unresectable CRLM (117). Patients were divided into three groups with each receiving mitomycin C alone, mitomycin C plus gemcitabine, or mitomycin C plus irinotecan and followed by embolization with starch microspheres. The authors reported that 1-year and 2-year survival rates were 62% and 28% respectively with no significant difference among the patient groups (117). A study by Gruber-Rouh et al. involved 564 patients who were infused with mitomycin C, gemcitabine, irinotecan or cisplatin depending on the prior systemic chemotherapy regimen (**Table 7**) (123). For instance, patients treated with systemic FOLFOX or FOLFIRI were treated with mitomycin alone. Embolization was performed with iodized oil and starch microspheres. The study reported survival of 14.3 months from the start of first cTACE (123).

**Table 7:** Studies describing the role of TACE in CRLM.

Study	Study design	Country/Region	Sample Size	Patients	Follow up/Inclusion period	Results	Additional data
Maraj et al. (121) 2023	Retrospective study	Canada	120	328 procedures of irinotecan-eluting microspheres TACE was performed in unresectable CRLM with <75% hepatic parenchymal disease, limited extrahepatic tumor burden and previous locoregional treatment.	Included patients between 2012 to 2020	Technical success rate was 85%; Median OS of 12.7 months; The OS improved if the patient has prior ablation (P<0.05), <25% hepatic tumor burden (P<0.001), and previously resected primary disease (P<0.05)	5% intraprocedural adverse events including groin hematoma without pseudoaneurysm, periprocedural pain and hepatic artery dissection; 6% post-procedural adverse events including post embolic cholecystitis, perforated gastric ulcer, bleeding duodenal ulcer and biloma.
Vogl et al. (122) 2018	Retrospective study	Germany	452	Total: 452 patients with CRLM unresponsive to systemic chemotherapy; TACE as palliative option: 233 patients; TACE followed by ablation as neoadjuvant therapy: 219 patients	Included patients between 2001 and 2015	OS and PFS in palliative group were 12.6 and 5.9 months respectively and in neoadjuvant group was 25.8 and 10.8 months respectively.	Extrahepatic metastases in both palliative and neoadjuvant group; Tumor number, location, average size of metastases in neoadjuvant group.
Gruber-Rouh et al. (123)2013	Retrospective study	Germany	564	564 patients underwent TACE; Mean number of sessions:6	Included patients between 1999 and 2011	Partial response: 16.7%; Stable disease: 48.2%; Progressive disease: 16.7%; 1-, 2-, and 3-year survival rates: 62%, 28%, and 7% respectively; Median survival from the start of TACE: 14.3 months	Predictors of survival: Indication of TACE and initial tumor response
Nishiofuku et al. (124) 2013	Prospective trial	Japan	24	24 patients treated with FOLFOX prior to TACE	Phase I patient recruitment from February 2008 to July 2008; Phase II patient recruitment from September 2008 to January 2010; Mean follow up duration was 17.4 months	Tumor response rate: 61.1%; Median hepatic PFS: 8.8 months; OS: 21.1 months	Grade 3 thrombocytopenia: 12.5%; Grade 3 AST elevation: 33.3%; Grade 3 ALT elevation: 12.5%; Grade 3 hyponatremia: 8.3%; Grade 3 cholecystitis: 4.2%
Albert et al. (120) 2011	Retrospective study	United States	121	121 patients were treated with TACE comprising cisplatin, mitomycin C, doxorubicin, ethiodized oil and polyvinyl alcohol particles	Included patients between 1992 and 2008	Partial response: 2%; Stable disease: 41%; Progressive disease: 57%; Median time to disease progression: 5 months; Median survival: 27 months from development of hepatic metastases and 9 months from chemoembolization; Survival was better when cTACE was performed prior to third line systemic chemotherapy	
Muller et al. (125) 2007	Prospective study	Germany	66	66 patients; 5-FU and GM-CSF infusion followed by embolization with Melphalan, lipiodol, and gelfoam; 54% of patients received prior systemic chemotherapy	–	Complete response: 1%; partial response: 42.4%; Stable disease: 18.2%; No response: 12.1%; Two-year survival: 66%; Time to progression: 8 months	Almost all patients experienced self-limiting side effects such as upper abdominal pain, vomiting and leukopenia
Wasser et al., 2005 (126)	Randomized prospective trial	Germany	21	21 patients with CRLM patients treated with TACE	Total follow up duration was 12-18 weeks	Median survival was 13.8 months; therapeutic response in three patients; progression free interval of 5.8 months	

TACE, Trans- arterial radioembolization; CRLM, Colorectal liver metastases; OS, Overall survival; PFS, Progression free survival; FOLFOX, 5-fluorouracil and oxaliplatin; AST, Aspartate transaminase; ALT, Alanine transaminase; 5-FU, 5-Fluorouracil; GM-CSF, Granulocyte monocyte- colony stimulating factor.

Vogl et al. studied on patients treated with cTACE as a palliative or a neoadjuvant option (**Table 7**) (122). The cTACE was followed by ablation in the neoadjuvant group. All the patients were refractory to prior systemic chemotherapy. Vogl et al. reported significant improvement in OS and PFS in palliative (12.6 and 5.9 months, respectively) and neoadjuvant (25.8 and 10.8 months, respectively) groups (122). The presence of extrahepatic metastases was described as the significant factor for OS and PFS in both palliative and neoadjuvant groups (122). Vogl et al. concluded that cTACE was effective in unresectable advanced CRLM and further improves survival, if followed by ablation (122). Nishiofuku et al. studied the efficacy of TACE with cisplatin powder and degradable starch microspheres (DSM) and a reported tumor response rate in 61.1% of patients (124). They also reported the median OS, PFS, and hepatic-PFS as 21.1 months, 5.8 months, and 8.8 months (124). However, majority of patients became eligible for surgical resection post-TACE, which might overestimate the OS benefit of TACE. The authors studied the tumor response rate in wild-type and mutated KRAS tumors to be around 75% and 66.7%, respectively (124). The study concluded that cisplatin, at a dose of 80 mg/m2 with the DSM, can provide a high tumor response rate and prolonged survival time for patients with unresectable CRLM refractory to FOLFOX systemic chemotherapy (124). Short embolization effect and good tumor response are the two main advantages of DSM-TACE over conventional TACE (127). In summary, all the described studies demonstrate that cTACE is a feasible treatment modality in patients who are unresponsive to conventional therapy.

The TACE in combination with RFA is studied to improve the survival and outcomes in single HCC lesion >5 cm and multiple HCC lesions >3 cm (128). The same has also been applied in CRLM by Faiella et al., who discovered a positive impact on the patient survival (129). However, the data is limited as the protocol for TACE is quite different from RFA. Regular TACE protocol is for widespread CRLM, while targeted TACE, along with RFA, can be used for focal metastases (128).

#### 4.1.2 DEBIRI-TACE

A current area of research involves the use of irinotecan drug-eluting beads (DEBIRI-TACE) to treat CRLM. The initial results of a Phase II clinical trial comprising 20 patients reported an 80% response rate with reduction of contrast enhancement of treated tumors following treatment with irinotecan drug-eluting beads [37]. Similarly, Aliberti et al. reported 78% tumor response rate at three months in a phase II study comprising 82 patients (130). All the patients had at least two failed systemic chemotherapy lines. The study also described the OS and PFS as 25 months and 8 months respectively (130). Martin et al. studied the efficacy of DEBIRI in patients refractory to oxaliplatin- and irinotecan-based systemic chemotherapy. The study concluded that DEBIRI was safe with minimal complications and 75% tumor response rate (131). This promising treatment for patients with colorectal metastases merits further study both as a salvage agent and potentially in combination with systemic chemotherapy. Fiorentini et al. compared the efficacy of FOLFIRI and DEBIRI-TACE (132). Median OS was longer for DEBIRI-TACE group (22 vs. 15 months). In addition, DEBIRI-TACE group had better quality of life (8 vs. 3 months) and objective tumor response (69% vs. 20%) (132). However, the study was limited by the omission of bevacizumab, oxaliplatin, panitumumab or cetuximab in the standard care of treatment (132). Martin et al. overcame this limitation by comparing DEBIRI plus systemic FOLFOX and bevacizumab with systemic FOLFOX plus bevacizumab alone (133). The study observed a significantly greater response rate in DEBIRI-FOLFOX arm compared to FOLFOX/bevacizumab arm at the end of 2 months (78% vs. 54%) and 6 months (76% vs. 60%) (133). Th significant tumor downsizing was observed in DEBIRI-FOLFOX arm than the comparison arm (35% vs. 16%) (133). The median PFS of 15.3 months was reported in DEBIRI-FOLFOX arm and 7.6 months in FOLFOX/bevacizumab arm (133). Nonetheless, the study by Martin et al. did not demonstrate improvement in OS compared to cTACE studies that excluded systemic chemotherapy (**Table 8**) (133). Recently, a systematic review by Akinwande et al. included 13 studies comprising a total of 850 patients (135). The weighted average PFS and OS were 8.1 months and 16.8 months respectively (135).

**Table 8:** Studies describing the role of DEBIRI-TACE in CRLM.

Study	Study design	Country/Region	Sample size	Patient characteristics	Follow up/Inclusion period	Results
Szemitko et al. (134) 2021	Retrospective study	Poland	52	52 patients underwent 202 DEBIRI-TACE	Included the patients between 2016 and 2019	Median survival: 13 months; 1-year survival: 63%; 2-year survival: 33%; Significant complications: 7.4%; PES: 51%;
Akinwande et al. (135) 2017	Systematic review	United States	850	13 studies with a total of 850 patients treated with systemic chemotherapy	Included patients until 2016	Average all-grade toxicity: 35.2%; Average response rate: 56.2% and 51.1% according to RECIST and modified RECIST/EASL response criteria; PFS: 8.1 months; OS: 16.8 months.
Martin et al. (133) 2015	Randomized control trial	United States	70	70 patients randomized to DEBIRI/FOLFOX group and FOLFOX/bevacizumab group	Median follow up of 19 months (range 17-38 months)	DEBIRI/FOLFOX vs. FOLFOX/bevacizumab: Grade 3/4 adverse events- 54% vs. 46%; Overall response rate: 78% vs. 54% at 2 months and 76% vs. 60% at 6 months; Tumor downsizing: 35% vs 16%; Median PFS: 15.3 months vs. 7.6 months (P=0.18).
Fiorentini et al. (132) 2012	Prospective study	Italy	74	74 patients randomized to FOLFIRI and DEBIRI-TACE	Included patients presenting between 2006 and 2008; Median follow up at 50 months	Median survival for DEBIRI and FOLFIRI: 22 vs. 15 months; PFS: 7 vs. 4 months; Quality of life: 8 vs. 3 months
Martin et al. (131) 2009	Prospective study	United States, Canada, Europe, and Australia	55	55 patients treated with DEBIRI-TACE with 2 as the median number of treatments per patient	Included patients between 2007 and2008	Median DFS and OS were 247 days and 343 days respectively; Downstaged disease in 10% of patients; Response rate at 6 and 12 months was 66% and 75%, respectively; Predictors of OS: extrahepatic disease and extent of prior chemotherapy

DEBIRI, irinotecan drug-eluting beads; TACE, Trans-arterial chemoembolization; PFS, progression free survival; OS, Overall survival; FOLFOX, 5-fluorouracil and oxaliplatin; DFS, Disease free survival.

The most common complications following TACE procedure include post-embolization syndrome (PES) (15-90%), cholecystitis, and hepatic insufficiency (134, 136). Complications such as segmental biliary dilatation, thrombocytopenia, leukopenia, hepatic artery thrombosis, embolus migration are less common (134). The etiology of PES is not entirely determined but several theories have been proposed including hepatic capsular distention, tumor necrosis, hepatic ischemia, anti-inflammatory response to chemotherapeutic medications and gallbladder infarction (136, 137). Paye et al. studied that the PES following TACE is due to injury to the non-tumoral hepatic cells (138). Risk factors for the adverse effects include complete flow stasis during embolization, lack of pre-treatment with lidocaine, infusion of > 100 mg of DEBIRI, bilirubin > 2 ug/dl, with > 50% liver involvement, and achievement of complete stasis (131). Hence, patients with extrahepatic metastases, tumor burden of >70% liver parenchyma, increased bilirubin levels (> 3mg/dl), renal dysfunction (serum creatinine, > 2 mg/dl), and complete portal venous thrombosis are usually excluded from TACE (123).

DEB-TACE has certain limitations including (i) inability to identify the beads in real-time which in turn prevents the visualization of intraoperative precise delivery and post-operative effects (ii) as the DEBs load only positively charged chemotherapeutic medications, the options of drugs are restricted (139). Hence, new drug carriers are being studied to overcome the limitations. Iodine-containing and superparamagnetic iron oxide- containing microspheres are studied to visualize on the X-ray and MR imaging respectively.

### 4.2 TARE

**Figure 3:**
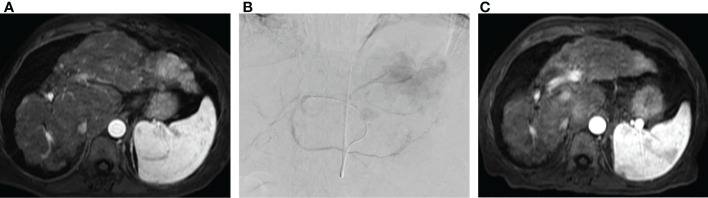
Radioembolization as salvage therapy. **(A)** Pre-procedure MRI in patient with metastatic colorectal cancer to the liver following three lines of chemotherapy demonstrating multifocal metastatic disease involving the left hepatic lobe. **(B)** Transradial radioembolization of the left hepatic lobe with Yttrium-90 resin microspheres *via* a replaced left hepatic artery arising from a left gastric artery. **(C)** Post procedure MRI with interval reduction in size and enhancement of left hepatic lobe tumor.

Guidelines support TARE as a treatment option in patients with CRLM who are refractory to ≥ 2 lines of systemic chemotherapy (**Figure 3**) (category 2A and Grade B recommendation as per European Society for Medical Oncology and National Comprehensive Cancer Network, respectively) (57, 140, 141). The application of TARE as a second-line therapy in unresectable CRLM refractory to first-line systemic chemotherapy require endorsement from further studies. Ideal candidates for Y90-TARE shall be ≥ 18 years old, Eastern Cooperative Oncology group (ECOG) score ≤ 2, serum bilirubin < 3 mg/dl, serum creatinine < 2 mg/dl, and with adequate lung function (140). Mulcahy et al. reported tumor response rate of 40.3% in unresectable CRLM when exposed to a median dose of 118Gy (**Table 9**) (148). The MORE study included 606 patients with CRLM who had two lines of prior systemic chemotherapy. The study reported OS of 9.6 months (144). Hickey et al. reported OS of 10.6 months in their study which involved 531 patients who were refractory to prior systemic chemotherapy or locoregional therapies (143). Absence of extrahepatic metastases, <25% tumor burden, albumin > 3 g/dl, good performance status and receipt of < 2 chemotherapeutic medications are the independent predictors of survival (143). In a prospective study by Helmberger et al. involving 1027 patients who underwent Y90-TARE for primary or metastatic hepatic tumors, the authors reported the OS of 9.8 months in CRLM (150). Wu et al. compared the survival outcomes with Y90-TARE in right versus left sided primary tumor location. They observed that patients with right sided primary tumors had decreased OS compared to left sided primary tumors (5.4 vs. 6.2 months; P=0.03) (151). However, no significant difference in hepatic PFS, tumor response and disease progression were observed (151). Lahti et al. studied the KRAS status as the prognostic factor in unresectable CRLM who underwent Y-90 TARE. They reported that median OS was greater in patients with KRAS wild-type genes than mutant genes (9.5 months vs. 4.8 months; P= 0.04) (152). The KRAS status, carcinoembryonic antigen levels, and Child-Pugh class were found to be the prognostic factors for OS (152). Narsinh et al. described the importance of hepatopulmonary shunting as a prognostic indicator of survival in their study of 606 patients who underwent Y90-TARE for CRLM. They reported that increased liver shunt fraction (LSF) indicated worse prognosis in CRLM. The LSF > 10% was associated with reduced survival rate compared to LSF < 10% (6.9 months vs. 10 months; HR: 1.60; P<0.001) (153).

**Table 9:** Studies describing the application of TARE in CRLM.

Study	Study design	Country/region	Sample size	Patient characteristics	Follow up/Inclusion period	Results	Additional data
Kalva et al. (142) 2017	Retrospective study	United States	45	45 patients with CRLM, who are unresponsive to chemotherapy	Included patients between 2005 to 2011	Technical success rate: 100%; Partial response: 2%; Stable disease (71%); Progressive disease (13%); PET response rate: 46%; Median survival: 186 days	Grade-3 toxicities: 13%; PET response was the independent predictor of OS; OS in PET responsive and non-responsive patients: 317 days vs. 163 days respectively.
Hickey et al. (143) 2016	Retrospective study	United States	531	531 patients who underwent radioembolization of CRLM	Included patients between 2001 and 2014	Median OS: 10.6 months; Median OS for patients who received three chemotherapeutics was shorter than those who received ≤ 2 chemotherapeutics (9.2 vs. 14.7 months)	Adverse events: Fatigue- 55%; Abdominal pain- 34%; Nausea- 19%; Grade 3/4 hyperbilirubinemia- 13%. Independent predictors of survival: Performance status, < 25% tumor burden, no extrahepatic metastases, albumin > 3 g/dl, and no more than two lines of chemotherapy.
Kennedy et al. (144) 2015	Retrospective study	United States	606	606 patients, with a prior history of two lines of chemotherapy, who underwent radioembolization for CRLM	Included patients between 2002 and 2011	Median survival following 2^nd^ -, 3^rd^ -, and 4^th^ - line chemotherapy was 13, 9, and 8.1 months respectively.	Garde ≥ 3 adverse events: Abdominal pain- 6.1%; Fatigue- 5.5%; Hyperbilirubinemia- 5.4%; Ascites- 3.6%; Gastrointestinal ulceration- 1.7%. Independent variables for survival: Stage of tumor, tumor to treated liver ratio, LFTs, leukocytes and prior history of chemotherapy.
Saxena et al. (145) 2014	Systematic review	Australia	979	20 studies with a total of 979 patients who failed atleast 3 lines of chemotherapy and underwent radioembolization	Included the studies performed before 2012	Complete radiological response: 0%; partial response: 31%; stable disease: 40.5%; OS: 12 months	Acute toxicity: 11-100%; Factors associated with poor survival: ≥ 3 lines of chemotherapy, extrahepatic disease, poor radiological response and extensive liver disease
Evans et al. (146) 2010	Retrospective study	Australia	140	140 patients with CRLM who are unresponsive to chemotherapy and underwent radioembolization	Included patients between 2006 to 2009	OS: 7.9 months;	Minor complications in the form of abdominal pain, nausea and vomiting.
Cianni et al. (147) 2009	Retrospective study	Italy	41	Patients with CRLM who are unresponsive to chemotherapy and underwent radioembolization	Included patients between 2005 and 2008	Complete response: 4.8%; partial response: 41.5%; Stable disease: 36.2%; Progressive disease: 19.5%; CEA reduced from 4.2 ug/L before treatment to 2.1 ug/L after treatment; Technical success rate: 98%; Median survival: 354 days; PFS: 279 days	Hepatic failure: 2%; Grade-2 gastritis: 4%; Grade-2 cholecystitis: 2%
Mulcahy et al. (148) 2009	Prospective study	United States	72	Patients with unresectable CRLM who ultimately underwent radioembolization	Included patients between 2003 and 2007	Tumor response rate: 40.3%; PET response rate: 77%; OS from the date of hepatic metastases: 34.6 months; OS from first Y90 treatment: 14.5 months; Patients with ECOG status 0 had a median survival of 42.8 months and 23.5 months from the date of hepatic metastases and Y90 treatment, respectively.	Fatigue (61%), nausea (21%), abdominal pain (25%), grade 3 & 4 bilirubin toxicities (12.6%).
Kennedy et al. (149) 2006	Prospective study	United States	208	Unresectable CRLM refractory to Oxaliplatin and Irinotecan	Included patients between 2002 and 2005; Median follow-up: 13 months; Median survival: 10.5 month in responders and 4.5 months in non-responders	CT partial response rate: 35%, PET response rate: 91%; CEA reduced by 70%	Nausea (9-10%), abdominal pain (11-13%), grade 2 & 3 bilirubin toxicity (3-4.5%), grade 2 & 3 ALP toxicity (20-20.5%)

CRLM, Colorectal liver metastases; PET, Positron emission tomography; OS, Overall survival; LFT, Liver function tests; CEA, Carcinoembryonic antigen; PFS, progression free survival; ECOG, Eastern cooperative oncology group; CT, Computed tomography.

Dendy et al. studied the survival predictive biomarkers in patients who underwent Y90-TARE for CRLM (140). They described that low tumor burden, sufficient calculated Y90 dose, increased albumin, and low ECOG score are the pre-interventional biomarkers which indicate favorable outcome (140). Likewise, after the procedure, decreased tumor burden, reduced tumor glycolysis, radiological tumor response and reduced expression of surviving, p53, Bcl-2 are indicative of favorable outcome (140). Irrespective of timing of biomarker evaluation, the increased HMGB1(High mobility group box 1), nucleosome expression, increased carcinoembryonic antigen, CA 19-9, CYFRA 21-1 (Cytokeratin 19 fragment), lactate dehydrogenase, aspartate transaminase, choline esterase, gamma glutamyl transferase, alkaline phosphatase, amylase are the indicators of unfavorable response (140). Usually, Y90 radioembolization is safe with minor complications and post-embolization syndrome. Gastric ulceration (<5%), portal hypertension (<1%), radiation induced liver fibrosis (<4%), pancreatitis (<1%), biloma (<1%), cholecystitis (<1%), abscess formation (<1%), and radiation induced pneumonitis (<1%) are the few of reported complications secondary to radioembolization (154). The post-embolization syndrome can be observed in 50% of the patients within 2 weeks of the procedure. In contrast to post-embolic syndrome, it rarely requires patient hospitalization.

## 5 Other hepatic metastases

Very few studies have been performed on the interventional management of non-CRLM. In liver metastases secondary to gastric tumors, RFA is proven beneficial only in cases of single metastases limited to a single lobe and without extrahepatic disease (155). Combined systemic chemotherapy is also recommended in addition to RFA to prolong the OS (155). RFA in liver metastases secondary to breast cancer has also been studied to improve OS; however, the extrahepatic metastases (P=0.013) and age >60 years (P=0.025) are considered worse prognostic factors for OS (156). MWA has equal benefits to RFA and can be an alternate therapy in patients with liver metastases originating from ovarian, pancreatic, esophageal, and neuroendocrine neoplasms (157). Further broad studies are required for more data on patient outcomes and efficacy. Arterial interventions such as TACE with raltitrexed eluting beads are studied to be safe and efficient in hepatic metastases due to gastric adenocarcinoma (158). In contrast to CRLM, the focus of arterial interventions in neuroendocrine liver metastases (NELM) is on the controlling the endocrine secretions (159). NELM are hypervascular tumors, and the studies show that the embolization alone has good efficacy on patient outcomes, unlike colorectal metastases, requiring chemotherapeutic embolization (160, 161). Elf et al. demonstrated that the NELM has optimal response rates to embolization therapies compared to SIRT (162). Other than CRLM and NELM, the literature is limited to other hepatic metastases. Saxena et al. studied that SIRT in chemoresistant hepatic metastases due to breast cancer has improved 24-month survival rates to 39% (163). Despite this, prospective trials on optimal patient selection and survival data are necessary for further validation.

## 6 Future directions

The combination of immunotherapy and targeted ablation is a new revolutionizing concept based on enhanced exposure of the tumor antigen. Ablated and dead tumor cells release tumor antigens into the bloodstream which augments the T-cell response, enhancing the efficacy of immunotherapy (164). Both the pro-inflammatory cytokines such as IL-6, and the anti-inflammatory cytokines, such as IL-10, get elevated after the ablation procedure. So far, cryoablation has been proven to induce a higher (4.6 fold) IL-10 release compared to heat-based techniques such as RFA (1,7 fold) and MWA (1.2 fold) (165, 166). Shi et al. reported that the PD-L1-PD-1 axis inhibits the T-cell response; hence monoclonal antibodies against the PD-1 are used to increase the feasibility of an anti-tumor immune response (167). The stronger T-cell response, robust anti-tumor immunity, and improved survival rates were observed in mice after combining anti-PD1 monoclonal antibodies with an ablation procedure (167). Likely, the TACE procedure triggers tissue hypoxia and the release of vascular endothelial growth factor, which could be used as the target for bevacizumab and tyrosine kinase inhibitors. Tumor-associated macrophages (TAMs) are responsible for tumor nurture and metastasization by inducing the epithelial-to-mesenchymal transition (EMT) and vascular disruption. Current studies are targeting TGF-beta signaling pathway, which is responsible for the EMT. The collagen triple helix repeat containing 1 (CTHRC1) is secreted by the colorectal cancer cells, stabilizing the TGF-beta signaling and activation. Studies show that the monoclonal antibodies against CTHRC1 combined with PD-1/PD-L1 blockade have led to the shrinkage of CRLM (168). Similarly, strategies targeting the TAMs reprogramming, depletion, and inhibition were studied (169). However, stronger validations are not yet provided due to the heterogenous behavior of the TAMs.

## 7 Conclusion

Tremendous evolution has occurred over the last two decades in the locoregional interventional therapies for CRLM. Surgical resection is the curative treatment for patients with CRLM. In case of unresectable tumors or non-surgical candidates, evaluation for ablation is recommended. Transarterial therapies are indicated as a salvage therapy and Y90-TARE is the FDA approved therapy for CRLM. DEBIRI-TACE or cTACE is considered in patients with progressive liver disease after Y90-TARE.

## Author contributions

SV, PS, SK, NO and SPK have contributed equally to this work. All authors contributed to the article and approved the submitted version.

## References

[1] Piawah S Venook AP. Targeted therapy for colorectal cancer metastases: a review of current methods of molecularly targeted therapy and the use of tumor biomarkers in the treatment of metastatic colorectal cancer. Cancer (2019) 125(23):4139–47. doi: 10.1002/cncr.32163 31433498

[2] Zhou H Liu Z Wang Y Wen X Amador EH Yuan L. Colorectal liver metastasis: molecular mechanism and interventional therapy. Signal Transduct Target Ther (2022) 7(1):70. doi: 10.1038/s41392-022-00922-2 35246503 PMC8897452

[3] Dekker E Tanis PJ Vleugels JLA Kasi PM Wallace MB. Colorectal cancer. Lancet (2019) 394(10207):1467–80. doi: 10.1016/S0140-6736(19)32319-0 31631858

[4] Riihimäki M Hemminki A Sundquist J Hemminki K. Patterns of metastasis in colon and rectal cancer. Sci Rep (2016) 6(1):1–9. doi: 10.1038/srep29765 27416752 PMC4945942

[5] Van Cutsem E Oliveira J. Advanced colorectal cancer: ESMO clinical recommendations for diagnosis, treatment and follow-up. Ann Oncol (2009) 20:iv61–iv3. doi: 10.1093/annonc/mdp130 19454465

[6] Siegel RL Miller KD Fedewa SA Ahnen DJ Meester RGS Barzi A. Colorectal cancer statistics, 2017. CA Cancer J Clin (2017) 67(3):177–93. doi: 10.3322/caac.21395 28248415

[7] Vatandoust S Price TJ Karapetis CS. Colorectal cancer: metastases to a single organ. World J Gastroenterol (2015) 21(41):11767. doi: 10.3748/wjg.v21.i41.11767 26557001 PMC4631975

[8] Osterlund P Salminen T Soveri LM Kallio R Kellokumpu I Lamminmäki A. Repeated centralized multidisciplinary team assessment of resectability, clinical behavior, and outcomes in 1086 Finnish metastatic colorectal cancer patients (RAXO): a nationwide prospective intervention study. Lancet Reg Health Eur (2021) 3:100049. doi: 10.1016/j.lanepe.2021.100049 34557799 PMC8454802

[9] Leporrier J Maurel J Chiche L Bara S Segol P Launoy G. A population-based study of the incidence, management and prognosis of hepatic metastases from colorectal cancer. J Br Surg (2006) 93(4):465–74. doi: 10.1002/bjs.5278 16523446

[10] Van Tilborg A Meijerink M Sietses C Van Waesberghe J Mackintosh M Meijer S. Long-term results of radiofrequency ablation for unresectable colorectal liver metastases: a potentially curative intervention. Br J Radiol (2011) 84(1002):556–65. doi: 10.1259/bjr/78268814 PMC3473628 21159807

[11] Al-Sharif E Simoneau E Hassanain M. Portal vein embolization effect on colorectal cancer liver metastasis progression: lessons learned. World J Clin Oncol (2015) 6(5):142–6. doi: 10.5306/wjco.v6.i5.142 PMC4600188 26468450

[12] Andreou A Aloia TA Brouquet A Vauthey J-N. Recent advances in the curative treatment of colorectal liver metastases. Gastrointest Cancer Res: GCR (2011) 4(4 Suppl 1):S2. 22368730 PMC3283006

[13] Fernandez FG Drebin JA Linehan DC Dehdashti F Siegel BA Strasberg SM. Five-year survival after resection of hepatic metastases from colorectal cancer in patients screened by positron emission tomography with f-18 fluorodeoxyglucose (FDG-PET). Ann Surg (2004) 240(3):438. doi: 10.1097/01.sla.0000138076.72547.b1 15319715 PMC1356434

[14] Abdalla EK Vauthey J-N Ellis LM Ellis V Pollock R Broglio KR. Recurrence and outcomes following hepatic resection, radiofrequency ablation, and combined resection/ablation for colorectal liver metastases. Ann Surg (2004) 239(6):818. doi: 10.1097/01.sla.0000128305.90650.71 15166961 PMC1356290

[15] Wei AC Greig PD Grant D Taylor B Langer B Gallinger S. Survival after hepatic resection for colorectal metastases: a 10-year experience. Ann Surg Oncol (2006) 13(5):668–76. doi: 10.1245/ASO.2006.05.039 16523369

[16] Adam R Avisar E Ariche A Giachetti S Azoulay D Castaing D. Five-year survival following hepatic resection after neoadjuvant therapy for nonresectable colorectal [liver] metastases. Ann Surg Oncol (2001) 8(4):347–53. doi: 10.1007/s10434-001-0347-3 11352309

[17] Deschamps F Ronot M Gelli M Durand-Labrunie J Tazdait M Hollebecque A. Interventional radiology for colorectal liver metastases. Curr Colorectal Cancer Rep (2020) 16(2):29–37. doi: 10.1007/s11888-020-00449-0

[18] Raoux L Maulat C Mokrane F-Z Fares N Suc B Muscari F. Impact of the strategy for curative treatment of synchronous colorectal cancer liver metastases. J Visceral Surg (2020) 157(4):289–99. doi: 10.1016/j.jviscsurg.2019.10.007 32089468

[19] Xing M Kooby DA El-Rayes BF Kokabi N Camacho JC Kim HS. Locoregional therapies for metastatic colorectal carcinoma to the liver–an evidence-based review. J Surg Oncol (2014) 110(2):182–96. doi: 10.1002/jso.23619 24760444

[20] Fairchild AH White SB. Decision making in interventional oncology: intra-arterial therapies for metastatic colorectal cancer-Y90 and chemoembolization. Semin Intervent Radiol (2017) 34(2):87–91. doi: 10.1055/s-0037-1601854 28579675 PMC5453773

[21] Mahnken AH Pereira PL de Baère T. Interventional oncologic approaches to liver metastases. Radiology (2013) 266(2):407–30. doi: 10.1148/radiol.12112544 23362094

[22] Makuuchi M Thai BL Takayasu K Takayama T Kosuge T Gunvén P. Preoperative portal embolization to increase safety of major hepatectomy for hilar bile duct carcinoma: a preliminary report. Surgery (1990) 107(5):521–7. 2333592

[23] de Graaf W van den Esschert JW van Lienden KP van Gulik TM. Induction of tumor growth after preoperative portal vein embolization: is it a real problem? Ann Surg Oncol (2009) 16(2):423–30. doi: 10.1245/s10434-008-0222-6 19050974

[24] Mueller L Hillert C Möller L Krupski-Berdien G Rogiers X Broering DC. Major hepatectomy for colorectal metastases: is preoperative portal occlusion an oncological risk factor? Ann Surg Oncol (2008) 15(7):1908–17. doi: 10.1245/s10434-008-9925-y 18459005

[25] Shindoh J Tzeng CW Aloia TA Curley SA Zimmitti G Wei SH. Optimal future liver remnant in patients treated with extensive preoperative chemotherapy for colorectal liver metastases. Ann Surg Oncol (2013) 20(8):2493–500. doi: 10.1245/s10434-012-2864-7 PMC3855465 23377564

[26] Ribero D Abdalla EK Madoff DC Donadon M Loyer EM Vauthey JN. Portal vein embolization before major hepatectomy and its effects on regeneration, resectability and outcome. Br J Surg (2007) 94(11):1386–94. doi: 10.1002/bjs.5836 17583900

[27] Abdalla EK Barnett CC Doherty D Curley SA Vauthey JN. Extended hepatectomy in patients with hepatobiliary malignancies with and without preoperative portal vein embolization. Arch Surg (2002) 137(6):675–80. doi: 10.1001/archsurg.137.6.675 12049538

[28] Wen XD Xiao L. Associating liver partition and portal vein ligation for staged hepatectomy in the treatment of colorectal cancer liver metastases. World J Gastrointest Surg (2021) 13(8):814–21. doi: 10.4240/wjgs.v13.i8.814 PMC8394386 34512905

[29] Pamecha V Levene A Grillo F Woodward N Dhillon A Davidson BR. Effect of portal vein embolisation on the growth rate of colorectal liver metastases. Br J Cancer (2009) 100(4):617–22. doi: 10.1038/sj.bjc.6604872 PMC2653734 19209170

[30] Collin Y Paré A Belblidia A Létourneau R Plasse M Dagenais M. Portal vein embolization does not affect the long-term survival and risk of cancer recurrence among colorectal liver metastases patients: a prospective cohort study. Int J Surg (2019) 61:42–7. doi: 10.1016/j.ijsu.2018.11.029 30537548

[31] Coco D Leanza S. Associating liver partition and portal vein ligation for staged hepatectomy (ALPPS) in colorectal liver metastases: review of the literature. Clin Exp Hepatol (2021) 7(2):125–33. doi: 10.5114/ceh.2021.106521 PMC8284168 34295978

[32] Dueland S Yaqub S Syversveen T Carling U Hagness M Brudvik KW. Survival outcomes after portal vein embolization and liver resection compared with liver transplant for patients with extensive colorectal cancer liver metastases. JAMA Surg (2021) 156(6):550–7. doi: 10.1001/jamasurg.2021.0267 PMC8014205 33787838

[33] Huiskens J Olthof PB van der Stok EP Bais T van Lienden KP Moelker A. Does portal vein embolization prior to liver resection influence the oncological outcomes - a propensity score matched comparison. Eur J Surg Oncol (2018) 44(1):108–14. doi: 10.1016/j.ejso.2017.09.017 29126672

[34] Ironside N Bell R Bartlett A McCall J Powell J Pandanaboyana S. Systematic review of perioperative and survival outcomes of liver resections with and without preoperative portal vein embolization for colorectal metastases. HPB (Oxford) (2017) 19(7):559–66. doi: 10.1016/j.hpb.2017.03.003 28438427

[35] Giglio MC Giakoustidis A Draz A Jawad ZAR Pai M Habib NA. Oncological outcomes of major liver resection following portal vein embolization: a systematic review and meta-analysis. Ann Surg Oncol (2016) 23(11):3709–17. doi: 10.1245/s10434-016-5264-6 27272106

[36] Hoekstra LT van Lienden KP Doets A Busch OR Gouma DJ van Gulik TM. Tumor progression after preoperative portal vein embolization. Ann Surg (2012) 256(5):812–7. doi: 10.1097/SLA.0b013e3182733f09 23095626

[37] Simoneau E Aljiffry M Salman A Abualhassan N Cabrera T Valenti D. Portal vein embolization stimulates tumour growth in patients with colorectal cancer liver metastases. HPB (Oxford) (2012) 14(7):461–8. doi: 10.1111/j.1477-2574.2012.00476.x PMC3384876 22672548

[38] Pamecha V Glantzounis G Davies N Fusai G Sharma D Davidson B. Long-term survival and disease recurrence following portal vein embolisation prior to major hepatectomy for colorectal metastases. Ann Surg Oncol (2009) 16(5):1202–7. doi: 10.1245/s10434-008-0269-4 19130138

[39] Kokudo N Tada K Seki M Ohta H Azekura K Ueno M. Proliferative activity of intrahepatic colorectal metastases after preoperative hemihepatic portal vein embolization. Hepatology (2001) 34(2):267–72. doi: 10.1053/jhep.2001.26513 11481611

[40] Geschwind JFH Salem R Carr BI Soulen MC Thurston KG Goin KA. Yttrium-90 microspheres for the treatment of hepatocellular carcinoma. Gastroenterology (2004) 127(5):S194–205. doi: 10.1053/j.gastro.2004.09.034 15508085

[41] Dawson LA Normolle D Balter JM McGinn CJ Lawrence TS Ten Haken RK. Analysis of radiation-induced liver disease using the Lyman NTCP model. Int J Radiat Oncol Biol Physics (2002) 53(4):810–21. doi: 10.1016/S0360-3016(02)02846-8 12095546

[42] Raval M Bande D Pillai AK Blaszkowsky LS Ganguli S Beg MS. Yttrium-90 radioembolization of hepatic metastases from colorectal cancer. Front Oncol (2014) 4:120. doi: 10.3389/fonc.2014.00120 25120951 PMC4110696

[43] Kalva SP Thabet A Wicky S. Recent advances in transarterial therapy of primary and secondary liver malignancies. Radiographics (2008) 28(1):101–17. doi: 10.1148/rg.281075115 18203933

[44] Chiu AM Savoor R Gordon AC Riaz A Sato KT Hohlastos E. Yttrium-90 radiation segmentectomy in oligometastatic secondary hepatic malignancies. J Vasc Interv Radiol (2023) 34(3):362–8. doi: 10.1016/j.jvir.2022.12.021 36526074

[45] Nebelung H Wolf T Bund S Radosa CG Plodeck V Grosche-Schlee S. Radioembolization versus portal vein embolization for contralateral liver lobe hypertrophy: effect of cirrhosis. Abdom Radiol (NY) (2021) 46(8):4046–55. doi: 10.1007/s00261-021-03048-1 PMC8286933 33779787

[46] Vouche M Lewandowski RJ Atassi R Memon K Gates VL Ryu RK. Radiation lobectomy: time-dependent analysis of future liver remnant volume in unresectable liver cancer as a bridge to resection. J Hepatol (2013) 59(5):1029–36. doi: 10.1016/j.jhep.2013.06.015 PMC5085290 23811303

[47] Teo JY Allen JC Jr. Ng DC Choo SP Tai DW Chang JP. A systematic review of contralateral liver lobe hypertrophy after unilobar selective internal radiation therapy with Y90. HPB (Oxford) (2016) 18(1):7–12. doi: 10.1016/j.hpb.2015.07.002 26776845 PMC4750235

[48] Garlipp B de Baere T Damm R Irmscher R van Buskirk M Stübs P. Left-liver hypertrophy after therapeutic right-liver radioembolization is substantial but less than after portal vein embolization. Hepatology (2014) 59(5):1864–73. doi: 10.1002/hep.26947 24259442

[49] Edeline J Lenoir L Boudjema K Rolland Y Boulic A Le Du F. Volumetric changes after (90)y radioembolization for hepatocellular carcinoma in cirrhosis: an option to portal vein embolization in a preoperative setting? Ann Surg Oncol (2013) 20(8):2518–25. doi: 10.1245/s10434-013-2906-9 23494107

[50] Kurilova I Pompa V Guerrero R Tapias MA Calatayud MD Fondevila C. (90)Y-radioembolization after failed portal vein embolization for colorectal liver metastases: a case report. Cardiovasc Intervent Radiol (2020) 43(8):1232–6. doi: 10.1007/s00270-020-02537-y 32514612

[51] Liebl M Pedersoli F Zimmermann M Schulze-Hagen M Truhn D Sieben P. Induction of contralateral hepatic hypertrophy by unilobar yttrium-90 transarterial radioembolization versus portal vein embolization: an animal study. J Vasc Interv Radiol (2021) 32(6):836–42.e2. doi: 10.1016/j.jvir.2021.01.281 33689835

[52] Fernández-Ros N Silva N Bilbao JI Iñarrairaegui M Benito A D'Avola D. Partial liver volume radioembolization induces hypertrophy in the spared hemiliver and no major signs of portal hypertension. HPB (Oxford) (2014) 16(3):243–9. doi: 10.1111/hpb.12095 PMC3945850 23530966

[53] Mima K Beppu T Chikamoto A Miyamoto Y Nakagawa S Kuroki H. Hepatic resection combined with radiofrequency ablation for initially unresectable colorectal liver metastases after effective chemotherapy is a safe procedure with a low incidence of local recurrence. Int J Clin Oncol (2013) 18(5):847–55. doi: 10.1007/s10147-012-0471-z 22940848

[54] Sasaki K Margonis GA Andreatos N Kim Y Wilson A Gani F. Combined resection and RFA in colorectal liver metastases: stratification of long-term outcomes. J Surg Res (2016) 206(1):182–9. doi: 10.1016/j.jss.2016.06.098 27916360

[55] Petre EN Sofocleous CT Solomon SB. Ablative and catheter-directed therapies for colorectal liver and lung metastases. Hematol Oncol Clin North Am (2015) 29(1):117–33. doi: 10.1016/j.hoc.2014.09.007 25475575

[56] Puijk RS Dijkstra M van den Bemd BAT Ruarus AH Nieuwenhuizen S Geboers B. Improved outcomes of thermal ablation for colorectal liver metastases: a 10-year analysis from the prospective Amsterdam CORE registry (AmCORE). Cardiovasc Intervent Radiol (2022) 45:1074–89. doi: 10.1007/s00270-022-03152-9 PMC9307533 35585138

[57] Tsitskari M Filippiadis D Kostantos C Palialexis K Zavridis P Kelekis N. The role of interventional oncology in the treatment of colorectal cancer liver metastases. Ann Gastroenterol (2019) 32(2):147. doi: 10.20524/aog.2019.0338 30837787 PMC6394269

[58] Rhim H Goldberg SN Dodd GD Solbiati L Lim HK Tonolini M. Essential techniques for successful radio-frequency thermal ablation of malignant hepatic tumors. Radiographics (2001) 21(suppl_1):S17–35. doi: 10.1148/radiographics.21.suppl_1.g01oc11s17 11598245

[59] Solbiati L Ahmed M Cova L Ierace T Brioschi M Goldberg SN. Small liver colorectal metastases treated with percutaneous radiofrequency ablation: local response rate and long-term survival with up to 10-year follow-up. Radiology (2012) 265(3):958–68. doi: 10.1148/radiol.12111851 23091175

[60] Kei SK Rhim H Choi D Lee WJ Lim HK Kim Y-s. Local tumor progression after radiofrequency ablation of liver tumors: analysis of morphologic pattern and site of recurrence. Am J Roentgenol (2008) 190(6):1544–51. doi: 10.2214/AJR.07.2798 18492905

[61] Wang C-Z Yan G-X Xin H Liu Z-Y. Oncological outcomes and predictors of radiofrequency ablation of colorectal cancer liver metastases. World J Gastrointest Oncol (2020) 12(9):1044. doi: 10.4251/wjgo.v12.i9.1044 33005297 PMC7509997

[62] de Baere T Deschamps F Briggs P Dromain C Boige V Hechelhammer L. Hepatic malignancies: percutaneous radiofrequency ablation during percutaneous portal or hepatic vein occlusion. Radiology (2008) 248(3):1056–66. doi: 10.1148/radiol.2483070222 18632532

[63] Lu DS Raman SS Limanond P Aziz D Economou J Busuttil R. Influence of large peritumoral vessels on outcome of radiofrequency ablation of liver tumors. J Vasc Interv Radiol (2003) 14(10):1267–74. doi: 10.1097/01.RVI.0000092666.72261.6B 14551273

[64] Nielsen K van Tilborg AA Meijerink MR Macintosh MO Zonderhuis BM de Lange ES. Incidence and treatment of local site recurrences following RFA of colorectal liver metastases. World J Surg (2013) 37(6):1340–7. doi: 10.1007/s00268-013-1997-6 23494086

[65] Curley SA Marra P Beaty K Ellis LM Vauthey JN Abdalla EK. Early and late complications after radiofrequency ablation of malignant liver tumors in 608 patients. Ann Surg (2004) 239(4):450. doi: 10.1097/01.sla.0000118373.31781.f2 15024305 PMC1356249

[66] Kim KH Yoon YS Yu CS Kim TW Kim HJ Kim PN. Comparative analysis of radiofrequency ablation and surgical resection for colorectal liver metastases. J Korean Surg Society (2011) 81(1):25–34. doi: 10.4174/jkss.2011.81.1.25 PMC3204557 22066097

[67] Gillams AR Lees WR. Five-year survival following radiofrequency ablation of small, solitary, hepatic colorectal metastases. J Vasc Interv Radiol (2008) 19(5):712–7. doi: 10.1016/j.jvir.2008.01.016 18440460

[68] Wang X Sofocleous CT Erinjeri JP Petre EN Gonen M Do KG. Margin size is an independent predictor of local tumor progression after ablation of colon cancer liver metastases. Cardiovasc Interv Radiol (2013) 36(1):166–75. doi: 10.1007/s00270-012-0377-1 PMC4122121 22535243

[69] Elias D Baton O Sideris L Matsuhisa T Pocard M Lasser P. Local recurrences after intraoperative radiofrequency ablation of liver metastases: a comparative study with anatomic and wedge resections. Ann Surg Oncol (2004) 11(5):500–5. doi: 10.1245/ASO.2004.08.019 15078636

[70] Xie X Jiang C Peng Z Liu B Hu W Wang Y. Local recurrence after radiofrequency ablation of hepatocellular carcinoma: treatment choice and outcome. J Gastrointest Surg (2015) 19(8):1466–75. doi: 10.1007/s11605-015-2850-z 26014717

[71] Pathak S Jones R Tang J Parmar C Fenwick S Malik H. Ablative therapies for colorectal liver metastases: a systematic review. Colorectal Dis (2011) 13(9):e252–e65. doi: 10.1111/j.1463-1318.2011.02695.x 21689362

[72] Filippiadis DK Velonakis G Kelekis A Sofocleous CT. The role of percutaneous ablation in the management of colorectal cancer liver metastatic disease. Diagnost (Basel) (2021) 11(2):308. doi: 10.3390/diagnostics11020308 PMC7918461 33672993

[73] Ierardi AM Floridi C Fontana F Chini C Giorlando F Piacentino F. Microwave ablation of liver metastases to overcome the limitations of radiofrequency ablation. La Radiol Medica (2013) 118(6):949–61. doi: 10.1007/s11547-013-0968-1 23892957

[74] Gravante G Ong SL Metcalfe MS Strickland A Dennison AR Lloyd DM. Hepatic microwave ablation: a review of the histological changes following thermal damage. Liver Int (2008) 28(7):911–21. doi: 10.1111/j.1478-3231.2008.01810.x 18564212

[75] Spiers HV Lancellotti F de Liguori Carino N Pandanaboyana S Frampton AE Jegatheeswaran S. Irreversible electroporation for liver metastases from colorectal cancer: a systematic review. Cancers (2023) 15(9):2428. doi: 10.3390/cancers15092428 37173895 PMC10177346

[76] Nieuwenhuizen S Dijkstra M Puijk RS Geboers B Ruarus AH Schouten EA. Microwave ablation, radiofrequency ablation, irreversible electroporation, and stereotactic ablative body radiotherapy for intermediate size (3-5 cm) unresectable colorectal liver metastases: a systematic review and meta-analysis. Curr Oncol Rep (2022) 24(6):793–808. doi: 10.1007/s11912-022-01248-6 35298796 PMC9054902

[77] Torres-Jiménez J Esteban-Villarrubia J Ferreiro-Monteagudo R Carrato A. Local treatments in the unresectable patient with colorectal cancer metastasis: a review from the point of view of the medical oncologist. Cancers (Basel) (2021) 13(23):5938. doi: 10.3390/cancers13235938 34885047 PMC8656541

[78] Scheffer HJ Nielsen K van Tilborg AA Vieveen JM Bouwman RA Kazemier G. Ablation of colorectal liver metastases by irreversible electroporation: results of the COLDFIRE-I ablate-and-resect study. Eur Radiol (2014) 24(10):2467–75. doi: 10.1007/s00330-014-3259-x 24939670

[79] Meijerink MR Ruarus AH Vroomen LGPH Puijk RS Geboers B Nieuwenhuizen S. Irreversible electroporation to treat unresectable colorectal liver metastases (COLDFIRE-2): a phase II, two-center, single-arm clinical trial. Radiology (2021) 299(2):470–80. doi: 10.1148/radiol.2021203089 33724066

[80] Schicho A Niessen C Haimerl M Wiesinger I Stroszczynski C Beyer LP. Long-term survival after percutaneous irreversible electroporation of inoperable colorectal liver metastases. Cancer Manag Res (2019) 11:317–22. doi: 10.2147/CMAR.S182091 PMC6312065 30643457

[81] Filippiadis D Mauri G Marra P Charalampopoulos G Gennaro N De Cobelli F. Percutaneous ablation techniques for renal cell carcinoma: current status and future trends. Int J Hyperthermia (2019) 36(2):21–30. doi: 10.1080/02656736.2019.1647352 31537160

[82] Crocetti L de Baére T Pereira PL Tarantino FP. CIRSE standards of practice on thermal ablation of liver tumours. Cardiovasc Intervent Radiol (2020) 43(7):951–62. doi: 10.1007/s00270-020-02471-z 32382856

[83] Ruers T Van Coevorden F Punt CJ Pierie J-PE Borel-Rinkes I Ledermann JA. Local treatment of unresectable colorectal liver metastases: results of a randomized phase II trial. JNCI: J Natl Cancer Institute (2017) 109(9):djx015. doi: 10.1093/jnci/djx015 PMC5408999 28376151

[84] Kazemier G. Unresectable colorectal cancer liver metastases treated by intraoperative radiofrequency ablation with or without resection (Br J surg 2012; 99: 558–565). Br J Surg (2012) 99(4):566. doi: 10.1002/bjs.8724 22396054

[85] Ruers T Punt C Van Coevorden F Pierie J Borel-Rinkes I Ledermann J. Radiofrequency ablation combined with systemic treatment versus systemic treatment alone in patients with non-resectable colorectal liver metastases: a randomized EORTC intergroup phase II study (EORTC 40004). Ann Oncol (2012) 23(10):2619–26. doi: 10.1093/annonc/mds053 PMC3457746 22431703

[86] Lee SL Bassetti MF Rusthoven CG. The role of stereotactic body radiation therapy in the management of liver metastases. Semin Radiat Oncol (2023) 33(2):181–92. doi: 10.1016/j.semradonc.2022.11.008 36990635

[87] Mohamad I Barry A Dawson L Hosni A. Stereotactic body radiation therapy for colorectal liver metastases. Int J Hyperthermia (2022) 39(1):611–9. doi: 10.1080/02656736.2021.1923836 35465818

[88] Tubin S Gupta S Grusch M Popper HH Brcic L Ashdown ML. Shifting the immune-suppressive to predominant immune-stimulatory radiation effects by SBRT-PArtial tumor irradiation targeting HYpoxic segment (SBRT-PATHY). Cancers (Basel) (2020) 13(1):50. doi: 10.3390/cancers13010050 33375357 PMC7795882

[89] Morris VK Kennedy EB Baxter NN Benson AB Cercek A Cho M. Treatment of metastatic colorectal cancer: ASCO guideline. J Clin Oncol (2023) 41(3):678–700. doi: 10.1200/JCO.22.01690 36252154 PMC10506310

[90] Petrelli F Comito T Barni S Pancera G Scorsetti M Ghidini A. Stereotactic body radiotherapy for colorectal cancer liver metastases: a systematic review. Radiother Oncol (2018) 129(3):427–34. doi: 10.1016/j.radonc.2018.06.035 29997034

[91] Lee J Shin IS Yoon WS Koom WS Rim CH. Comparisons between radiofrequency ablation and stereotactic body radiotherapy for liver malignancies: meta-analyses and a systematic review. Radiother Oncol (2020) 145:63–70. doi: 10.1016/j.radonc.2019.12.004 31923711

[92] Entezari P Gabr A Salem R Lewandowski RJ. Yttrium-90 for colorectal liver metastasis - the promising role of radiation segmentectomy as an alternative local cure. Int J Hyperthermia (2022) 39(1):620–6. doi: 10.1080/02656736.2021.1933215 35465813

[93] Vouche M Habib A Ward TJ Kim E Kulik L Ganger D. Unresectable solitary hepatocellular carcinoma not amenable to radiofrequency ablation: multicenter radiology-pathology correlation and survival of radiation segmentectomy. Hepatology (2014) 60(1):192–201. doi: 10.1002/hep.27057 24691943

[94] Dupré A Jones RP Diaz-Nieto R Fenwick SW Poston GJ Malik HZ. Curative-intent treatment of recurrent colorectal liver metastases: a comparison between ablation and resection. Eur J Surg Oncol (2017) 43(10):1901–7. doi: 10.1016/j.ejso.2017.08.008 28888801

[95] He X Zhang P Li Z Bi F Xu F Wang X. Curative-intent radiotherapy in patients with oligometastatic lesions from colorectal cancer: a single-center study. Med (Baltimore) (2018) 97(40):e12601. doi: 10.1097/MD.0000000000012601 PMC6200534 30290630

[96] Jia Z Wang C Paz-Fumagalli R Wang W. Radiation segmentectomy for hepatic malignancies: indications, devices, dosimetry, procedure, clinical outcomes, and toxicity of yttrium-90 microspheres. J Interv Med (2019) 2(1):1–4. doi: 10.1016/j.jimed.2019.05.001 34805860 PMC8562265

[97] Zerizer I Al-Nahhas A Towey D Tait P Ariff B Wasan H. The role of early ^18^F-FDG PET/CT in prediction of progression-free survival after ^90^Y radioembolization: comparison with RECIST and tumour density criteria. Eur J Nucl Med Mol Imag (2012) 39(9):1391–9. doi: 10.1007/s00259-012-2149-1 22644713

[98] Jongen JMJ Rosenbaum C Braat M van den Bosch M Sze DY Kranenburg O. Anatomic versus metabolic tumor response assessment after radioembolization treatment. J Vasc Interv Radiol (2018) 29(2):244–53.e2. doi: 10.1016/j.jvir.2017.09.024 29249594

[99] Sager S Akgün E Uslu-Beşli L Asa S Akovali B Sahin O. Comparison of PERCIST and RECIST criteria for evaluation of therapy response after yttrium-90 microsphere therapy in patients with hepatocellular carcinoma and those with metastatic colorectal carcinoma. Nucl Med Commun (2019) 40(5):461–8. doi: 10.1097/MNM.0000000000001014 30896544

[100] Shady W Kishore S Gavane S Do RK Osborne JR Ulaner GA. Metabolic tumor volume and total lesion glycolysis on FDG-PET/CT can predict overall survival after (90)Y radioembolization of colorectal liver metastases: a comparison with SUVmax, SUVpeak, and RECIST 1.0. Eur J Radiol (2016) 85(6):1224–31. doi: 10.1016/j.ejrad.2016.03.029 PMC5675072 27161074

[101] Shady W Sotirchos VS Do RK Pandit-Taskar N Carrasquillo JA Gonen M. Surrogate imaging biomarkers of response of colorectal liver metastases after salvage radioembolization using 90Y-loaded resin microspheres. AJR Am J Roentgenol (2016) 207(3):661–70. doi: 10.2214/AJR.15.15202 PMC5675077 27384594

[102] Kurilova I Bendet A Fung EK Petre EN Humm JL Boas FE. Radiation segmentectomy of hepatic metastases with y-90 glass microspheres. Abdom Radiol (NY) (2021) 46(7):3428–36. doi: 10.1007/s00261-021-02956-6 PMC9513941 33606062

[103] Padia SA Johnson GE Agopian VG DiNorcia J Srinivasa RN Sayre J. Yttrium-90 radiation segmentectomy for hepatic metastases: a multi-institutional study of safety and efficacy. J Surg Oncol (2021) 123(1):172–8. doi: 10.1002/jso.26223 32944980

[104] Meiers C Taylor A Geller B Toskich B. Safety and initial efficacy of radiation segmentectomy for the treatment of hepatic metastases​. J Gastrointest Oncol (2018) 9(2):311–5. doi: 10.21037/jgo.2017.11.02 PMC5934149 29755770

[105] Padia SA Kwan SW Roudsari B Monsky WL Coveler A Harris WP. Superselective yttrium-90 radioembolization for hepatocellular carcinoma yields high response rates with minimal toxicity. J Vasc Interv Radiol (2014) 25(7):1067–73. doi: 10.1016/j.jvir.2014.03.030 24837982

[106] Biederman DM Titano JJ Korff RA Fischman AM Patel RS Nowakowski FS. Radiation segmentectomy versus selective chemoembolization in the treatment of early-stage hepatocellular carcinoma. J Vasc Interv Radiol (2018) 29(1):30–7.e2. doi: 10.1016/j.jvir.2017.08.026 29169782

[107] Lewandowski RJ Gabr A Abouchaleh N Ali R Al Asadi A Mora RA. Radiation segmentectomy: potential curative therapy for early hepatocellular carcinoma. Radiology (2018) 287(3):1050–8. doi: 10.1148/radiol.2018171768 29688155

[108] Haber Z Lee EW Price M Wainberg Z Hecht JR Sayre J. Survival advantage of yttrium-90 radioembolization to systemic therapy in patients with hepatic metastases from colorectal cancer in the salvage setting: results of a matched pair study. Acad Radiol (2021) 28:S210–S7. doi: 10.1016/j.acra.2021.03.033 34099386

[109] Gibbs P Gebski V Van Buskirk M Thurston K Cade DN Van Hazel GA. Selective internal radiation therapy (SIRT) with yttrium-90 resin microspheres plus standard systemic chemotherapy regimen of FOLFOX versus FOLFOX alone as first-line treatment of non-resectable liver metastases from colorectal cancer: the SIRFLOX study. BMC Cancer (2014) 14:897. doi: 10.1186/1471-2407-14-897 25487708 PMC4289171

[110] Van Hazel GA Heinemann V Sharma NK Peeters M. SIRFLOX: randomized phase III trial comparing first-line mFOLFOX6 (plus or minus bevacizumab) versus mFOLFOX6 (plus or minus bevacizumab) plus selective internal radiation therapy in patients with metastatic colorectal cancer. J Clin Oncol -New York (2016) 34(15):1723–31. doi: 10.1200/JCO.2015.66.1181 26903575

[111] Dutton SJ Kenealy N Love SB Wasan HS Sharma RA. FOXFIRE protocol: an open-label, randomised, phase III trial of 5-fluorouracil, oxaliplatin and folinic acid (OxMdG) with or without interventional selective internal radiation therapy (SIRT) as first-line treatment for patients with unresectable liver-only or liver-dominant metastatic colorectal cancer. BMC Cancer (2014) 14:497. doi: 10.1186/1471-2407-14-497 25011439 PMC4107961

[112] Gibbs P Heinemann V Sharma NK Taieb J Ricke J Peeters M. Effect of primary tumor side on survival outcomes in untreated patients with metastatic colorectal cancer when selective internal radiation therapy is added to chemotherapy: combined analysis of two randomized controlled studies. Clin Colorectal Cancer (2018) 17(4):e617–e29. doi: 10.1016/j.clcc.2018.06.001 30033117

[113] Wasan HS Gibbs P Sharma NK Taieb J Heinemann V Ricke J. First-line selective internal radiotherapy plus chemotherapy versus chemotherapy alone in patients with liver metastases from colorectal cancer (FOXFIRE, SIRFLOX, and FOXFIRE-global): a combined analysis of three multicentre, randomised, phase 3 trials. Lancet Oncol (2017) 18(9):1159–71. doi: 10.1016/S1470-2045(17)30457-6 PMC5593813 28781171

[114] van Hazel G Heinemann V Sharma N Taieb J Ricke J Peeters M. Impact of primary tumour location on survival in patients with metastatic colorectal cancer receiving selective internal radiation therapy and chemotherapy as first-line therapy. Ann Oncol (2017) 28:iii152. doi: 10.1093/annonc/mdx302.005

[115] Garlipp B Gibbs P Van Hazel GA Jeyarajah R Martin RCG Bruns CJ. Secondary technical resectability of colorectal cancer liver metastases after chemotherapy with or without selective internal radiotherapy in the randomized SIRFLOX trial. Br J Surg (2019) 106(13):1837–46. doi: 10.1002/bjs.11283 PMC6899564 31424576

[116] Breedis C Young G. The blood supply of neoplasms in the liver. Am J Pathol (1954) 30(5):969. 13197542 PMC1942491

[117] Vogl TJ Zangos S Eichler K Yakoub D Nabil M. Colorectal liver metastases: regional chemotherapy *via* transarterial chemoembolization (TACE) and hepatic chemoperfusion: an update. Eur Radiol (2007) 17(4):1025–34. doi: 10.1007/s00330-006-0372-5 16944163

[118] Yamada R Sato M Kawabata M Nakatsuka H Nakamura K Takashima S. Hepatic artery embolization in 120 patients with unresectable hepatoma. Radiology (1983) 148(2):397–401. doi: 10.1148/radiology.148.2.6306721 6306721

[119] Gruber-Rouh T Marko C Thalhammer A Nour-Eldin NE Langenbach M Beeres M. Current strategies in interventional oncology of colorectal liver metastases. Br J Radiol (2016) 89(1064):20151060. doi: 10.1259/bjr.20151060 27164030 PMC5124876

[120] Albert M Kiefer MV Sun W Haller D Fraker DL Tuite CM. Chemoembolization of colorectal liver metastases with cisplatin, doxorubicin, mitomycin c, ethiodol, and polyvinyl alcohol. Cancer (2011) 117(2):343–52. doi: 10.1002/cncr.25387 20830766

[121] Maraj T Mirrahimi A Dey C. Survival of patients with colorectal liver metastases after transarterial chemoembolization using irinotecan-eluting microspheres: a single-center retrospective analysis comparing RECIST 1.1 and choi criteria. J Vasc Interv Radiol (2023). doi: 10.1016/j.jvir.2023.02.005 36775014

[122] Vogl TJ Lahrsow M Albrecht MH Hammerstingl R Thompson ZM Gruber-Rouh T. Survival of patients with non-resectable, chemotherapy-resistant colorectal cancer liver metastases undergoing conventional lipiodol-based transarterial chemoembolization (cTACE) palliatively versus neoadjuvantly prior to percutaneous thermal ablation. Eur J Radiol (2018) 102:138–45. doi: 10.1016/j.ejrad.2018.03.015 29685527

[123] Gruber-Rouh T Naguib NN Eichler K Ackermann H Zangos S Trojan J. Transarterial chemoembolization of unresectable systemic chemotherapy-refractory liver metastases from colorectal cancer: long-term results over a 10-year period. Int J Cancer (2014) 134(5):1225–31. doi: 10.1002/ijc.28443 23960002

[124] Nishiofuku H Tanaka T Matsuoka M Otsuji T Anai H Sueyoshi S. Transcatheter arterial chemoembolization using cisplatin powder mixed with degradable starch microspheres for colorectal liver metastases after FOLFOX failure: results of a phase I/II study. J Vasc Interv Radiol (2013) 24(1):56–65. doi: 10.1016/j.jvir.2012.09.010 23194749

[125] Müller H Nakchbandi V Chatzisavvidis I von Voigt C. Repetitive chemoembolization with melphalan plus intra-arterial immuno-chemotherapy within 5-fluorouracil and granulocyte-macrophage colony-stimulating factor (GM-CSF) as effective first- and second-line treatment of disseminated colorectal liver metastases. Hepatogastroenterology (2003) 50(54):1919–26. 14696433

[126] Wasser K Giebel F Fischbach R Tesch H Landwehr P. [Transarterial chemoembolization of liver metastases of colorectal carcinoma using degradable starch microspheres (Spherex): personal investigations and review of the literature]. Radiologe (2005) 45(7):633–43. doi: 10.1007/s00117-004-1061-5 15316615

[127] Vogl TJ Marko C Langenbach MC Naguib NNN Filmann N Hammerstingl R. Transarterial chemoembolization of colorectal cancer liver metastasis: improved tumor response by DSM-TACE versus conventional TACE, a prospective, randomized, single-center trial. Eur Radiol (2021) 31(4):2242–51. doi: 10.1007/s00330-020-07253-2 32960329

[128] Seager MJ Jakobs TF Sharma RA Bandula S. Combination of ablation and embolization for intermediate-sized liver metastases from colorectal cancer: what can we learn from treating primary liver cancer? Diagn Interv Radiol (2021) 27(5):677–83. doi: 10.5152/dir.2021.20520 PMC8480946 34318754

[129] Faiella E Calabrese A Santucci D de Felice C Pusceddu C Fior D. Combined trans-arterial embolization and ablation for the treatment of Large (>3 cm) liver metastases: review of the literature. J Clin Med (2022) 11(19):5576. doi: 10.3390/jcm11195576 36233437 PMC9571710

[130] Aliberti C Fiorentini G Muzzio PC Pomerri F Tilli M Dallara S. Trans-arterial chemoembolization of metastatic colorectal carcinoma to the liver adopting DC bead®, drug-eluting bead loaded with irinotecan: results of a phase II clinical study. Anticancer Res (2011) 31(12):4581–7. 22199334

[131] Martin RC Robbins K Tomalty D O'Hara R Bosnjakovic P Padr R. Transarterial chemoembolisation (TACE) using irinotecan-loaded beads for the treatment of unresectable metastases to the liver in patients with colorectal cancer: an interim report. World J Surg Oncol (2009) 7:80. doi: 10.1186/1477-7819-7-80 19886993 PMC2777901

[132] Fiorentini G Aliberti C Tilli M Mulazzani L Graziano F Giordani P. Intra-arterial infusion of irinotecan-loaded drug-eluting beads (DEBIRI) versus intravenous therapy (FOLFIRI) for hepatic metastases from colorectal cancer: final results of a phase III study. Anticancer Res (2012) 32(4):1387–95. 22493375

[133] Martin RC 2nd Scoggins CR Schreeder M Rilling WS Laing CJ Tatum CM. Randomized controlled trial of irinotecan drug-eluting beads with simultaneous FOLFOX and bevacizumab for patients with unresectable colorectal liver-limited metastasis. Cancer (2015) 121(20):3649–58. doi: 10.1002/cncr.29534 26149602

[134] Szemitko M Golubinska-Szemitko E Sienko J Falkowski A. Complications following irinotecan-loaded microsphere chemoembolization of colorectal metastatic liver lesions associated with hepatic-artery branch temporary stasis. Curr Oncol (2021) 28(3):2296–307. doi: 10.3390/curroncol28030211 PMC8293090 34203031

[135] Akinwande O Dendy M Ludwig JM Kim HS. Hepatic intra-arterial injection of irinotecan drug eluting beads (DEBIRI) for patients with unresectable colorectal liver metastases: a systematic review. Surg Oncol (2017) 26(3):268–75. doi: 10.1016/j.suronc.2017.05.003 28807246

[136] Dhand S Gupta R. Hepatic transcatheter arterial chemoembolization complicated by postembolization syndrome. Semin Intervent Radiol (2011) 28(2):207–11. doi: 10.1055/s-0031-1280666 PMC3193324 22654264

[137] Gonsalves CF Brown DB. Chemoembolization of hepatic malignancy. Abdom Imag (2009) 34(5):557–65. doi: 10.1007/s00261-008-9446-y 18668189

[138] Paye F Farges O Dahmane M Vilgrain V Flejou JF Belghiti J. Cytolysis following chemoembolization for hepatocellular carcinoma. Br J Surg (1999) 86(2):176–80. doi: 10.1046/j.1365-2168.1999.01014.x 10100782

[139] He Y Zhang Y Gong Y Zhang Z Xu T Tian L. Multimodal imaging of nano-assembled microspheres loaded with doxorubicin and cisplatin for liver tumor therapy. Front Bioeng Biotechnol (2022) 10:1024174. doi: 10.3389/fbioe.2022.1024174 36213082 PMC9539659

[140] Dendy MS Ludwig JM Kim HS. Predictors and prognosticators for survival with yttrium-90 radioembolization therapy for unresectable colorectal cancer liver metastasis. Oncotarget (2017) 8(23):37912–22. doi: 10.18632/oncotarget.16007 PMC5514961 28415671

[141] Wang DS Louie JD Sze DY. Evidence-based integration of yttrium-90 radioembolization in the contemporary management of hepatic metastases from colorectal cancer. Tech Vasc Interv Radiol (2019) 22(2):74–80. doi: 10.1053/j.tvir.2019.02.007 31079714

[142] Kalva SP Rana RS Liu R Rachamreddy N Dave B Sharma A. Yttrium-90 radioembolization as salvage therapy for liver metastases from colorectal cancer. Am J Clin Oncol (2017) 40(3):288–93. doi: 10.1097/COC.0000000000000151 25374143

[143] Hickey R Lewandowski RJ Prudhomme T Ehrenwald E Baigorri B Critchfield J. 90Y radioembolization of colorectal hepatic metastases using glass microspheres: safety and survival outcomes from a 531-patient multicenter study. J Nucl Med (2016) 57(5):665–71. doi: 10.2967/jnumed.115.166082 26635340

[144] Kennedy AS Ball D Cohen SJ Cohn M Coldwell DM Drooz A. Multicenter evaluation of the safety and efficacy of radioembolization in patients with unresectable colorectal liver metastases selected as candidates for (90)Y resin microspheres. J Gastrointest Oncol (2015) 6(2):134–42. doi: 10.3978/j.issn.2078-6891.2014.109 PMC4311082 25830033

[145] Saxena A Bester L Shan L Perera M Gibbs P Meteling B. A systematic review on the safety and efficacy of yttrium-90 radioembolization for unresectable, chemorefractory colorectal cancer liver metastases. J Cancer Res Clin Oncol (2014) 140(4):537–47. doi: 10.1007/s00432-013-1564-4 PMC11824097 24318568

[146] Evans KA Richardson MG Pavlakis N Morris DL Liauw W Bester L. Survival outcomes of a salvage patient population after radioembolization of hepatic metastases with yttrium-90 microspheres. J Vasc Interv Radiol (2010) 21(10):1521–6. doi: 10.1016/j.jvir.2010.06.018 20813542

[147] Cianni R Urigo C Notarianni E Saltarelli A Salvatori R Pasqualini V. Selective internal radiation therapy with SIR-spheres for the treatment of unresectable colorectal hepatic metastases. Cardiovasc Intervent Radiol (2009) 32(6):1179–86. doi: 10.1007/s00270-009-9658-8 19680720

[148] Mulcahy MF Lewandowski RJ Ibrahim SM Sato KT Ryu RK Atassi B. Radioembolization of colorectal hepatic metastases using yttrium-90 microspheres. Cancer (2009) 115(9):1849–58. doi: 10.1002/cncr.24224 19267416

[149] Kennedy AS Coldwell D Nutting C Murthy R Wertman DE Jr. Loehr SP. Resin 90Y-microsphere brachytherapy for unresectable colorectal liver metastases: modern USA experience. Int J Radiat Oncol Biol Phys (2006) 65(2):412–25. doi: 10.1016/j.ijrobp.2005.12.051 16690429

[150] Helmberger T Golfieri R Pech M Pfammatter T Arnold D Cianni R. Clinical application of trans-arterial radioembolization in hepatic malignancies in Europe: first results from the prospective multicentre observational study CIRSE registry for SIR-spheres therapy (CIRT). Cardiovasc Intervent Radiol (2021) 44(1):21–35. doi: 10.1007/s00270-020-02642-y 32959085 PMC7728645

[151] Wu V Li MD Goodwin JS Wehrenberg-Klee EP Zurkiya O Kalva SP. Yttrium-90 hepatic radioembolization for advanced chemorefractory metastatic colorectal cancer: survival outcomes based on right- versus left-sided primary tumor location. AJR Am J Roentgenol (2021) 217(5):1141–52. doi: 10.2214/AJR.20.25315 33594907

[152] Lahti SJ Xing M Zhang D Lee JJ Magnetta MJ Kim HS. KRAS status as an independent prognostic factor for survival after yttrium-90 radioembolization therapy for unresectable colorectal cancer liver metastases. J Vasc Interv Radiol (2015) 26(8):1102–11. doi: 10.1016/j.jvir.2015.05.032 26210240

[153] Narsinh KH Van Buskirk M Kennedy AS Suhail M Alsaikhan N Hoh CK. Hepatopulmonary shunting: a prognostic indicator of survival in patients with metastatic colorectal adenocarcinoma treated with (90)Y radioembolization. Radiology (2017) 282(1):281–8. doi: 10.1148/radiol.2016152100 PMC5207118 27440733

[154] Riaz A Lewandowski RJ Kulik LM Mulcahy MF Sato KT Ryu RK. Complications following radioembolization with yttrium-90 microspheres: a comprehensive literature review. J Vasc Interv Radiol (2009) 20(9):1121–30. doi: 10.1016/j.jvir.2009.05.030 19640737

[155] Hwang JE Kim SH Jin J Hong JY Kim MJ Jung SH. Combination of percutaneous radiofrequency ablation and systemic chemotherapy are effective treatment modalities for metachronous liver metastases from gastric cancer. Clin Exp Metastasis (2014) 31(1):25–32. doi: 10.1007/s10585-013-9606-5 23975154 PMC3892106

[156] Schullian P Johnston E Laimer G Putzer D Eberle G Scharll Y. Stereotactic radiofrequency ablation of breast cancer liver metastases: short- and long-term results with predicting factors for survival. Cardiovasc Intervent Radiol (2021) 44(8):1184–93. doi: 10.1007/s00270-021-02820-6 PMC8249280 33825059

[157] Vogl TJ Jaraysa Y Martin SS Gruber-Rouh T Savage RH Nour-Eldin NA. A prospective randomized trial comparing microwave and radiofrequency ablation for the treatment of liver metastases using a dual ablation system ─ the Mira study. Eur J Radiol Open (2022) 9:100399. doi: 10.1016/j.ejro.2022.100399 35155721 PMC8822176

[158] Bi Y Jiao D Wang Y Han X Ren J. Preliminary outcomes of raltitrexed eluting bead-transarterial chemoembolization using callispheres® beads for gastrointestinal adenocarcinoma liver metastasis. World J Surg Oncol (2022) 20(1):229. doi: 10.1186/s12957-022-02696-x 35821043 PMC9277920

[159] Barat M Cottereau AS Kedra A Dermine S Palmieri LJ Coriat R. The role of interventional radiology for the treatment of hepatic metastases from neuroendocrine tumor: an updated review. J Clin Med (2020) 9(7):2302. doi: 10.3390/jcm9072302 32698459 PMC7408651

[160] Fiorentini G Sarti D Nani R Aliberti C Fiorentini C Guadagni S. Updates of colorectal cancer liver metastases therapy: review on DEBIRI. Hepat Oncol (2020) 7(1):HEP16. doi: 10.2217/hep-2019-0010 32273974 PMC7137176

[161] Maire F Lombard-Bohas C O'Toole D Vullierme MP Rebours V Couvelard A. Hepatic arterial embolization versus chemoembolization in the treatment of liver metastases from well-differentiated midgut endocrine tumors: a prospective randomized study. Neuroendocrinology (2012) 96(4):294–300. doi: 10.1159/000336941 22507901

[162] Elf AK Andersson M Henrikson O Jalnefjord O Ljungberg M Svensson J. Radioembolization versus bland embolization for hepatic metastases from small intestinal neuroendocrine tumors: short-term results of a randomized clinical trial. World J Surg (2018) 42(2):506–13. doi: 10.1007/s00268-017-4324-9 PMC5762793 29167951

[163] Saxena A Kapoor J Meteling B Morris DL Bester L. Yttrium-90 radioembolization for unresectable, chemoresistant breast cancer liver metastases: a large single-center experience of 40 patients. Ann Surg Oncol (2014) 21(4):1296–303. doi: 10.1245/s10434-013-3436-1 24337647

[164] Dai Y Zhao W Yue L Dai X Rong D Wu F. Perspectives on immunotherapy of metastatic colorectal cancer. Front Oncol (2021) 11:659964. doi: 10.3389/fonc.2021.659964 34178645 PMC8219967

[165] Erinjeri JP Fine GC Adema GJ Ahmed M Chapiro J den Brok M. Immunotherapy and the interventional oncologist: challenges and opportunities-a society of interventional oncology white paper. Radiology (2019) 292(1):25–34. doi: 10.1148/radiol.2019182326 31012818 PMC6604797

[166] Adnan A Sheth RA Tam A. Oligometastatic disease in the liver: the role of interventional oncology. Br J Radiol (2022) 95(1138):20211350. doi: 10.1259/bjr.20211350 35230141 PMC9815735

[167] Shi L Chen L Wu C Zhu Y Xu B Zheng X. PD-1 blockade boosts radiofrequency ablation-elicited adaptive immune responses against tumor. Clin Cancer Res (2016) 22(5):1173–84. doi: 10.1158/1078-0432.CCR-15-1352 PMC4780056 26933175

[168] Zhang XL Hu LP Yang Q Qin WT Wang X Xu CJ. CTHRC1 promotes liver metastasis by reshaping infiltrated macrophages through physical interactions with TGF-β receptors in colorectal cancer. Oncogene (2021) 40(23):3959–73. doi: 10.1038/s41388-021-01827-0 33986509

[169] Gazzillo A Polidoro MA Soldani C Franceschini B Lleo A Donadon M. Relationship between epithelial-to-Mesenchymal transition and tumor-associated macrophages in colorectal liver metastases. Int J Mol Sci (2022) 23(24):16197. doi: 10.3390/ijms232416197 36555840 PMC9783529

